# Determination of the two-dimensional distributions of gold nanorods by multiwavelength analytical ultracentrifugation

**DOI:** 10.1038/s41467-018-07366-9

**Published:** 2018-11-21

**Authors:** Simon E. Wawra, Lukas Pflug, Thaseem Thajudeen, Carola Kryschi, Michael Stingl, Wolfgang Peukert

**Affiliations:** 1 Institute of Particle Technology (LFG), Friedrich-Alexander-Universität Erlangen-Nürnberg (FAU), Cauerstrasse 4, 91058 Erlangen, Germany; 2 Mathematical Optimization, Friedrich-Alexander-Universität Erlangen-Nürnberg (FAU), Cauerstrasse 11, 91058 Erlangen, Germany; 3 Interdisciplinary Center for Functional Particle Systems (FPS), Friedrich-Alexander-Universität Erlangen-Nürnberg (FAU), Haberstrasse 9a, 91058 Erlangen, Germany; 4 Department of Chemistry and Pharmacy and Interdisciplinary Center for Molecular Materials (ICMM), Friedrich-Alexander-Universität Erlangen-Nürnberg (FAU), Egerlandstr. 3, 91058 Erlangen, Germany; 5 Present Address: School of Mechanical Sciences, Indian Institute of Technology Goa, Ponda, 403401 India

## Abstract

Properties of nanoparticles are influenced by various parameters like size, shape or composition. Comprehensive high throughput characterization techniques are urgently needed to improve synthesis, scale up to production and make way for new applications of multidimensional particulate systems. In this study, we present a method for measuring two-dimensional size distributions of plasmonic nanorods in a single experiment. Analytical ultracentrifuge equipped with a multiwavelength extinction detector is used to record the optical and sedimentation properties of gold nanorods simultaneously. A combination of sedimentation and extinction properties, both depending on diameter and length of the dispersed nanorods, is used to measure two-dimensional distributions of gold nanorod samples. The length, diameter, aspect ratio, volume, surface and cross-sectional distributions can be readily obtained from these results. As the technique can be extended to other non-spherical plasmonic particles and can be used for determining relative amounts of particles of different shapes it provides complete and quantitative insights into particulate systems.

## Abstract (web summary)

Characterization of nanoparticles is a complex and important problem for the vast number of applications that require them. Here, the authors present a method to uncover the two-dimensional distribution of length and diameter of anisotropic nanoparticles like gold nanorods with a single measurement by combining spectroscopic and sedimentation data.

## Introduction

Plasmonic nanoparticles are quite unique since their optical properties are tunable by varying their composition, size, shape, structure, and environment^1,2^. The spectral properties of noble metal nanoparticles are governed by the localized surface plasmon resonance^3,4^. This unique feature allows them to serve as agents for cancer cell imaging, photothermal therapy, drug delivery and as sensitizer in cancer radiation therapy^5–8^. In particular, gold nanorods are commonly used in biomedicine^5–9^, sensors^8,10,11^, and nanooptics^12^. In line with increasing demands, scalable synthesis strategies for preparation of well-defined noble-metal nanoparticles remain a topic of high scientific interest^13,14^. In the case of gold nanorods, fine-tuning of their size and shape anisotropy is necessary to optimize their spectral properties for specific applications^6,15^. Fast and accurate size determination is therefore essential. Although numerous methods for quick and accurate characterization of spherical nanoparticles are known, an efficient analysis of nonspherical nanoparticles is still lacking. Quite often the morphology and size distributions of nanoparticles are examined by transmission electron microscopy (TEM), which is quite time intensive for counting a statistically relevant number of nanoparticles. Dynamic light scattering (DLS), which is commonly used for the characterization of colloidal nanoparticles, does not allow the measurement of size and aspect ratio as the effects of both translational and rotational diffusion of the nanoparticles need to be taken into account^16^. The use of depolarized DLS allows measuring both quantities and deducing a mean length but the determination of the aspect ratio remains a challenge. Therefore the analysis of two-dimensional (2D) distributions is beyond reach^17^. Small angle X-ray scattering (SAXS) has been used to study the growth mechanism of gold nanorods during synthesis^18^ allowing an in situ determination of mean lengths and diameters with high temporal resolution. However, deconvolution of the scattering profiles is mathematically ill-posed and often leads to ambiguous results even for one-dimensional (1D) distributions where a predefined mathematical form (e.g. lognormal) has to be assumed. The combination of SAXS with small angle neutron scattering (SANS) analysis can provide important background information for analytical ultracentrifugation (AUC) analysis: SAXS gives information on the core whereas SANS can provide the thickness of the stabilizing organic shell around the core as was shown, for instance for ZnO quantum dots^19,20^. Recently, Thajudeen et al. showed that the mean length and diameter of nanorods can be accurately measured using a combination of electrospray scanning mobility particle sizer and AUC^21^.

Methods to measure complete 2D distributions are quite rare. In principle, 2D distributions of particles can be obtained by image analysis and counting of SEM or TEM images^22^. However, this approach is extremely time-consuming since several thousand particles must be analyzed for proper statistical analysis. Among the few published data sets are mass-mobility diameter distributions measured by scanning mobility analysis^23^ and in particular, data obtained from 2D AUC analysis for size and density^24,25^. In the recent past, analysis of sedimentation and optical properties using AUC equipped with multiwavelength detector has been successfully implemented for spherical nanoparticles^26,27^. To the best of our knowledge, except microscopy-based techniques, there are no other methods that allow measuring the full 2D size distribution of plasmonic nanoparticles dispersed in a colloidal system. Information on shape anisotropy of gold nanoparticles can be deduced from facile VIS-NIR spectroscopy experiments as the spectral position of the longitudinal surface plasmon resonance band is strongly dependent on the surrounding medium and the aspect ratio distribution of the nanoparticles^2,28–33^. The correct modeling of optical spectra of gold nanorods has been the subject of intensive debate in the past. The Gans model^3^ describes the absorption and the scattering cross-section of ellipsoidal particles. In addition, it was also used for the study of rod-like nanoparticles^4,28,29^. Other methods such as the discrete dipole approximation or the finite element method (FEM) allow calculating the spectra of individual true rod-like particles. This high flexibility and precision comes at the cost of high computational effort and requires the full structural information including the influence of the crystalline structure of the gold nanorods, the end cap geometry^34–38^, the used optical constants of the material^30^ and the optical properties of the host medium. These boundary conditions limit the applicability of detailed FEM models since nanoparticulate samples inevitably exhibit polydispersity in terms of size, shape and internal structure and depend on the chemical environment including the amount of ligand being present at the surface. Therefore, the analysis of the optical spectra of a nanoparticle ensemble is limited to the determination of the aspect ratio distribution under the assumption of normal distributions or constant volume and can neither provide the full 2D distribution nor length and diameter distributions simultaneously. In contrast, the combination of hydrodynamic and spectral analysis by utilizing the multiwavelength analytical ultracentrifuge (MWL-AUC) has an immense potential for the advanced analysis of anisotropic plasmonic dispersions.

We demonstrate in this manuscript that a single experiment in an analytical ultracentrifuge equipped with a multiwavelength UV-VIS-NIR detector can be used to obtain full 2D size distributions of plasmonic anisotropic nanoparticles such as gold nanorods from which the 1D diameter, length, aspect ratio, volume surface and cross-sectional distributions can be readily obtained. In addition, it is also possible to determine the amount of fast sedimenting spherical gold particles in the nanorod samples. The proposed method is validated by numerical simulation and the values retrieved from the experimental measurements agree well with the dimensions of the nanorods measured by TEM. Additionally, the applicability of different optical models is discussed in the context of the method. It is noteworthy that the analysis does not rely on any prior knowledge of the shape of the 2D distribution or the number of modalities. This methodology can be extended to all nanoparticles of different morphologies exhibiting a characteristic signature of their size, shape or composition in their optical spectra.

## Results

### Theoretical considerations

AUC has already proven to be a powerful characterization technique for colloidal nanoparticles^39,40^. In the MWL-AUC, both the temporal and radial evolution of the nanoparticle concentration in a centrifugal field can be monitored and recorded using in situ UV−VIS−NIR spectroscopy^41–43^. This approach gives access to a three-dimensional data space covering sedimentation coefficient, wavelength and associated extinction. The sedimentation coefficient is the sedimentation velocity normalized by the applied centrifugal field and depends solely on material properties like mass/size, density and shape and solvent parameters.

For nonspherical nanoparticles, the sedimentation coefficient, as given by Eq. 1, depends on the mass *m*, frictional ratio $\frac{f}{{f_0}}$ (which is the ratio of the hydrodynamic diameter to the volume equivalent diameter *x*_V_) of the particle, solvent density *ρ*_s_, particle density *ρ*_P_ and solvent viscosity *η*.

1 $$s = \frac{{m \cdot (1 - \frac{{\rho _{\mathrm{s}}}}{{\rho _{\mathrm{P}}}})}}{{3\pi \eta \cdot \frac{f}{{f_0}} \cdot x_{\mathrm{V}}}}.$$

The influence of hydration and the ligand shell needs to be considered additionally^21^. Since the MWL-AUC can be used to obtain sedimentation coefficient distributions for different wavelengths, the sedimentation properties can be correlated with the optical properties of the sample. Therefore, the temporal evolution of the extinction is recorded for wavelengths between 200 and 1000 nm and can be used for simultaneous spectral and sedimentation analysis. Typically, several detector pixels are combined to provide wavelength intervals of roughly 1 nm. MWL-AUC has been successfully used for determining the size-dependent optical band gap of CdTe by extraction of optical spectra of individual species^44^, scattering corrections to volume cumulative distributions in dynamic rotor speed experiments^26^ and the investigation of protein−RNA interactions^45^.

To address the problem of size and shape determination for gold nanorods, we have developed a novel “optical back-coupling” (OBC) method for MWL-AUC by acquiring spectral information of particles depending on their sedimentation behavior. The optical spectra and the embedded geometrical information are then coupled with the corresponding sedimentation coefficient to access multidimensional information. In the case of gold nanorods, this involves the analysis of the optical absorption spectra in combination with the corresponding sedimentation coefficient data of only one single experiment.

The particle mass *m*, particle density *ρ*_P_ and volume equivalent particle diameter *x*_V_ can be described as a function of the length *l* and the diameter *d* of a nanorod along with the height *h* and the density of the ligand shell *ρ*_shell_. The frictional ratio can also be expressed as a function of the aspect ratio (length to diameter ratio of a nanorod)^46^. The details of these calculations can be found in Supplementary Note 1. As shown by Link et al.^28^ and Eustis et al.^29^, the Vis-NIR extinction spectra of gold nanorods depends on the aspect ratio distribution. Consequently, once the shell parameters are known, the aspect ratio distribution deduced from the Vis-NIR extinction spectra and the corresponding sedimentation coefficient distribution can be combined to derive the 2D size distribution. This approach requires the solution of Eq. 1 for distinct lengths and diameters which provides access to the dimensions of the dispersed nanorods.

Spectral and sedimentation data for the fast sedimenting gold nanorods were acquired using the dynamic rotor speed technique that is suitable for measuring broad distributions or high sedimentation coefficients at constant speed. Therefore, extinction data were recorded at a constant radial location relative to the rotor center. The temporal evolution of the extinction data then allows calculating sedimentation coefficient distributions for each wavelength, leading to a three-dimensional data space, where sedimentation coefficients and wavelength provide two dimensions, while the associated extinction values cover the third dimension. Extinction spectra can be extracted for arbitrary sedimentation coefficient intervals. We direct the attention of the readers to a recent publication that provides the technical details and the possibilities of using the experimental procedure (dynamic rotor speed experiment)^26^. Please note that the presented analysis does not inherently depend on the exact method of data acquisition.

As a precondition for the realized experiments, the effects of translational diffusion need to be negligible, while rotational diffusion should be high enough to prevent the possibility of preferential alignment of nanorods in a centrifugal field. The typical perpendicular rotational Péclet number with the corresponding diffusion coefficient *D*_⊥_^47^ for a radial position *r* and a rod of length *l*, ${\mathrm{Pe}}_{{\mathrm{rot}}, \bot } = \frac{{l \cdot s\omega ^2r}}{{D_ \bot }}$ is quite low (≈10^−18^) and the translational Péclet number for a measurement cell with sedimentation path *l*_AUC_ and the translational diffusion coefficient *D*_T_, ${\mathrm{Pe}}_{{\mathrm{trans}}} = \frac{{l_{{\mathrm{AUC}}} \cdot s\omega ^2r}}{{D_{\mathrm{T}}}}$ is sufficiently high (≈10^3^). The negligibly low value of the rotational Péclet number and the sufficiently high translational Péclet number ensure that the preconditions are satisfied.

### Optical back-coupling method

**Fig. 1:**
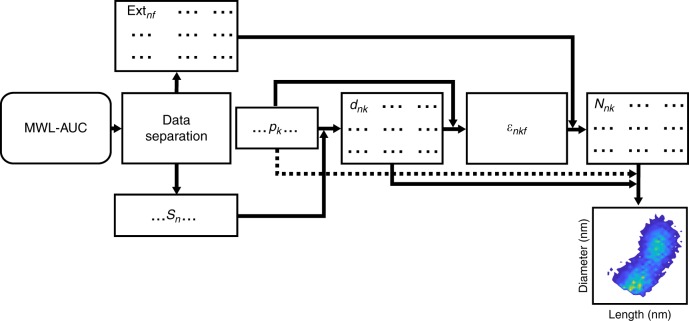
Information flow within the OBC method. The cumulative distribution at a fixed wavelength is separated in intervals of a constant height, then the corresponding spectrum and the mean sedimentation coefficient are deduced. Each spectrum is individually fitted for the number concentration ${{N}}$ of species of varying aspect ratio, length and diameter

The data analysis in the study is explained in detail using a flow chart as shown in Fig. 1. The normalized cumulative distribution at 480 nm was separated into a defined number of intervals, with each interval representing the same increment in extinction. The wavelength is chosen as the corresponding extinction value is not influenced by any plasmon resonance. This allows determining the corresponding extinction value Ext_*nf*_ in the sedimentation coefficient interval *n* for all available wavelengths *λ*_*f*_ separately and determining the corresponding center values of the sedimentation coefficient *s*_*n*_. In order to describe the optical spectra depending on the diameter and length of the gold nanorods, a linearly spaced array with different aspect ratios *p*_*k*_ (independent of the chosen sedimentation coefficient interval) is initialized. In the next step, the diameter *d*_*nk*_ is resolved for each aspect ratio *p*_*k*_ based on Eq. 1 using the mean sedimentation coefficient *s*_*n*_ of the respective interval. Since all nanoparticles contribute to the measured signal, Eq. 2 gives the resulting equality for the extracted spectrum Ext_*n*_ at wavelength *λ*_*f*_ including *N*_*nk*_, the number concentration of the nanoparticles, ${\it{\epsilon }}_{nkf}$the extinction cross-section of the particle with diameter *d*_*nk*_ and aspect ratio *p*_*k*_ and the optical path length *z*. After rearrangement of the extinction cross-sections in a matrix with dimensions of species *k* and wavelengths *λ*_*f*_, one can calculate the vector of number concentration *N*_*nk*_ for the *n*th-spectrum given in Eq. 3 by using a non-negative linear least-square solver.

2 $${\mathrm{Ext}}_{nf} = \mathop {\sum }\limits_k N_{nk} \cdot {\it{\epsilon }}_{nkf} \cdot z,$$

3 $$\left( {\begin{array}{*{20}{c}} {{\mathrm{Ext}}_{n1}} & \cdots & {{\mathrm{Ext}}_{nf{\max}}} \end{array}} \right) \\ = \left( {\begin{array}{*{20}{c}} {N_{n1}} & \cdots & {N_{nk{\max}}} \end{array}} \right)\left( {\begin{array}{*{20}{c}} {{\it{\epsilon }}_{n11}} & \cdots & {{\it{\epsilon }}_{n1f{\max}}} \\ \vdots & \ddots & \vdots \\ {{\it{\epsilon }}_{nk{\max}1}} & \cdots & {{\it{\epsilon }}_{nk{\max}f{\max}}} \end{array}} \right) \cdot z$$

In our analysis, only species with a theoretical peak maximum for the longitudinal surface plasmon resonance mode between the local spectral minimum at roughly 550 nm and a chosen maximum wavelength of 920 nm are considered. It has already been shown^28^ that the resonance peak for the transverse mode is less sensitive to the shape anisotropy. Finally, the number concentrations *N*_*nk*_ from all intervals/spectra are added together and classified according to their *d*_*nk*_ and *l*_*nk*_ to obtain the full 2D number distribution. From these, the distributions of diameter, length and all other related distributions can be calculated.

**Fig. 2:**
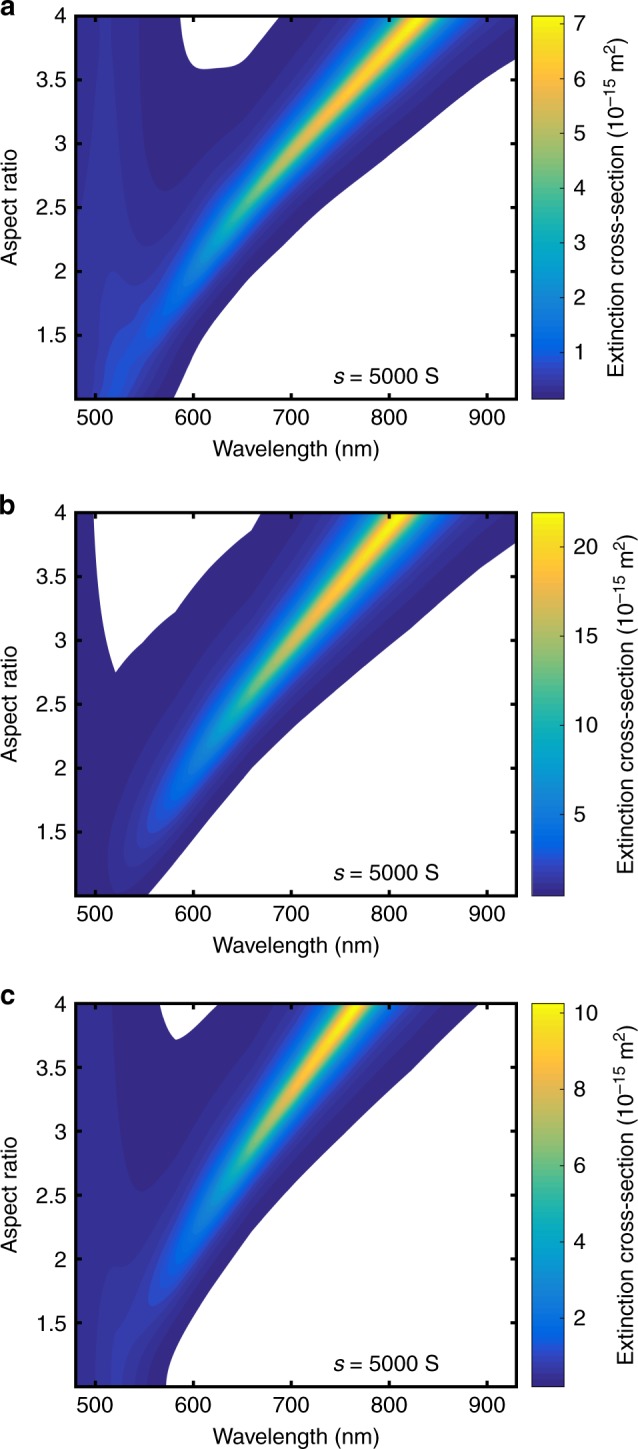
Influence of optical modeling on simulated gold nanorod spectra. Comparison of **a** FEM, **b** LP and **c** Gans model for nanorods with similar sedimentation coefficient but varying aspect ratio. While the LP model matches the resonance wavelength well, the Gans model gives better estimates for the absolute value of the cross-sections

As already discussed, the modeling of the optical properties of plasmonic nanoparticles is challenging, as the different models give different values for ${\it{\epsilon }}_{nkf}$. To find the best solution for the determination of true 2D distributions via the OBC method, we compare three different approaches. These include the classical Gans theory^2,29^ (see Supplementary Note 2) and an analytical longitudinal polarization (LP) model, which considers only polarization along the length axis of the nanorods^48^ (based on boundary element modeling), both allowing to adjust the optical properties of the surrounding medium. Additionally, we use a common orientationally averaged FEM model of nanoparticles in water. The particles’ geometry was either ellipsoidal (Gans) or spherical cylindrical using the same dielectric parameters^49^. Figure 2 shows extinction cross-sections for the different models. Differences in the resonance wavelength between sophisticated optical models and the Gans theory have already been discussed in published studies^35,36^. While the linewidth is similar for all models, the absolute value for the extinction cross-section deviates for the LP model^48^. This is a result of the missing orientation-averaging as only polarization along the length axis is included. Hence, the determination of the absolute concentration may be false; however, the relative concentration will still be sufficiently captured.

### Simulation

To test the performance of the methodology regardless of the chosen optical model, simulated data without random or systematic noise were evaluated first. A grid of 500 × 500 nanoparticles with a mean length and diameter of 45 nm and 20 nm with a standard deviation of 3 nm and 2 nm, respectively, was used. The lengths and diameters of the nanorods used in the study are assumed to be normally distributed. This distribution closely represents the distribution of CTAB sample 1, which is used in our experimental study. For each nanoparticle, the sedimentation coefficient and extinction spectra (as obtained by the different models) were calculated. The data were then rearranged and classified to mimic the results of an AUC experiment. For the analysis, the number of sedimentation coefficient intervals was chosen to be 50 within the range of 0.01−0.99 in the cumulative distribution. Additional Information on the effect of the number of chosen intervals and the corresponding solution space is provided in Supplementary Notes 4 and 5.

**Fig. 3:**
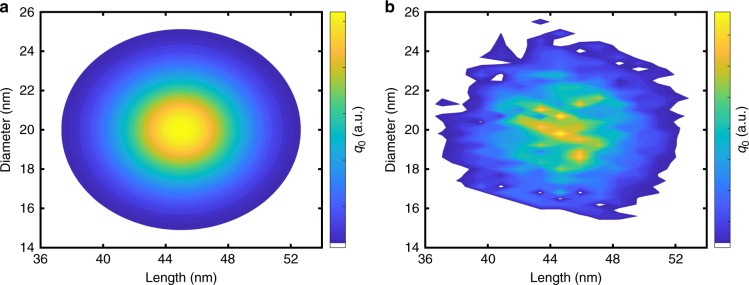
Two-dimensional size distributions obtained by simulation. Comparison of **a** input and **b** output distributions using the FEM model. Parameters of the ligand shell and the surrounding medium were assumed to be known

Figure 3 compares the input 2D size distribution with the retrieved distribution for the FEM model. For known shell parameters, the complete distribution can be accurately retrieved. Due to the separation in sedimentation coefficient intervals, the OBC method is rather stable against experimental random noise. However, the deconvolution of the spectra could be affected. In order to investigate this issue, Gaussian noise at different signal-to-noise-ratios was added to the spectra of every interval. Even though the shape of the distributions gets distorted to some extent, the results still provide information of excellent quality. Additional information is provided in Supplementary Note 7.

For an unknown nanoparticle sample, the analysis is more complicated as the parameters for the dielectric permittivity of the surrounding medium ${\it{\epsilon }}_{\mathrm{m}},$ the shell thickness *h* and shell density *ρ*_shell_ are unknown. Since the shell partially consists of ligands and adsorbed solvent molecules, the value of the shell density was chosen as the mean value of densities of the ligand and the solvent. A similar approach was successfully used in our recent study on the determination of the average length and diameter of nanorods in colloidal systems^21^. *ρ*_shell_ is kept constant, while the other two values are, in dependence on the optical model, determined for a certain ligand/nanoparticle system combination. In order to allow easier identification of deviations, one dimensional length, diameter and aspect ratio distributions are extracted from the 2D information.

As the LP and Gans models give analytical expressions of the extinction cross-section, ${\it{\epsilon }}_{\mathrm{m}}$ and *h* can be adjusted, while the FEM model allows only the adjustment of *h* due to the extensive computational demands of this model. The MATLAB^©^ function fminsearch, based on a simplex method^50^, was implemented in the evaluation code to minimize the root-mean-square deviation between the input distributions and the retrieved distributions (*p*, *l*, and *d*). The adjusted parameters ${\it{\epsilon }}_{\mathrm{m}}$ and *h* deviate less than 0.5% from the input values of the simulations for the LP and the Gans model, while the deviation of *h* is less than 2% for the FEM model. This means that under the assumption of correct optical modeling these parameters may be well adjusted to obtain the true 2D size distribution as well as the cumulative length and diameter distributions. Additionally, the 2D and 1D size, surface and volume distributions can be extracted (Supplementary Note 3).

### Experimental results

To test the performance of the analysis for distinct systems, three samples with CTAB and two samples with citrate as stabilizer and differing aspect ratios were analyzed. The first CTAB gold nanorod sample with the smallest aspect ratio was used to determine values for ${\it{\epsilon }}_{\mathrm{m}},h$ (Gans, LP model) or *h* (FEM) by using TEM measurements as a reference. A representative TEM image is given in Supplementary Figure 41.

**Fig. 4:**
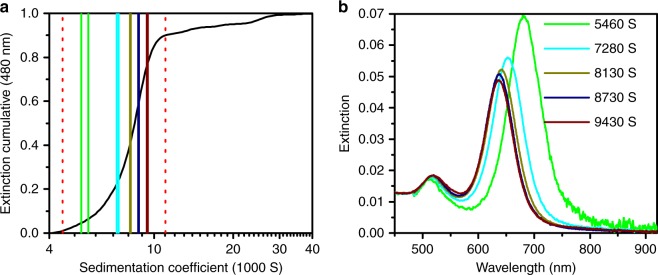
Acquired sedimentation and spectral data for the first CTAB sample. **a** Range of analysis (dotted red) and selected sedimentation coefficient intervals and **b** corresponding extracted spectra with mean sedimentation coefficients. The color of the intervals corresponds to the upper and lower boundary as well as to the extracted spectra. For reasons of clarity not every spectra or interval is shown. The interval length varies according to the cumulative distribution from 60 to 600 S, the extinction is based on the natural logarithm

For the analysis, 50 sedimentation coefficient intervals within 0.01−0.9 of the normalized cumulative distribution were used. 0.9 was used as the upper limit from the cumulative distribution as agglomerates are more likely to be present in the flat region of the distribution (see Fig. 4). The spectra show a clear correlation between the spectral position of the longitudinal surface plasmon resonance and the sedimentation coefficient. The best result for the cumulative aspect ratio and length and diameter distributions were obtained for the adjusted parameters $({\it{\epsilon }}_{\mathrm{m}},h)$ given in Table 1.

**Table 1:** List of adjusted parameters

Quantity and sample	Gans	LP	FEM
${\it{\epsilon }}_{\mathrm{m}}$ CTAB 1/—	2.26	1.96	—
$h$ CTAB 1/nm	4.2	4.1	4.1
${\it{\epsilon }}_{\mathrm{m}}$ citrate 1/—	2.07	1.84	—
$h$ citrate 1/nm	4.1	4.1	4.0

**Fig. 5:**
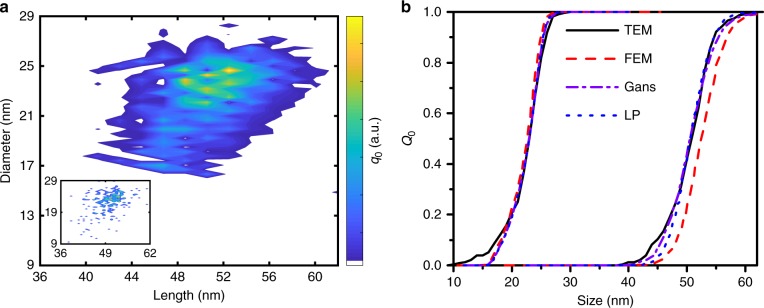
Resulting size distributions for the first CTAB sample. Comparison of **a** two-dimensional length and size distributions from AUC (Gans) and TEM statistics (inset with 230 TEM counted particles) and **b** size cumulative distributions derived from the model-based determination of the relevant parameters by sedimentation analysis

In a prior study^51^, we presented a comparison of AUC measurements with SAXS/SANS measurements, wherein the results confirmed that the shell thickness around nanoparticles can reliably be determined by AUC. The results obtained for the gold nanorods are similar to values found in literature^21,52–54^. The retrieved cumulative distributions and the 2D size distributions, as shown in Fig. 5, agree very well with the data obtained from TEM image analysis. Additionally, 2D size distributions for the LP and the FEM models and the corresponding aspect ratio distributions are shown in Supplementary Figures 25 and 26.

**Fig. 6:**
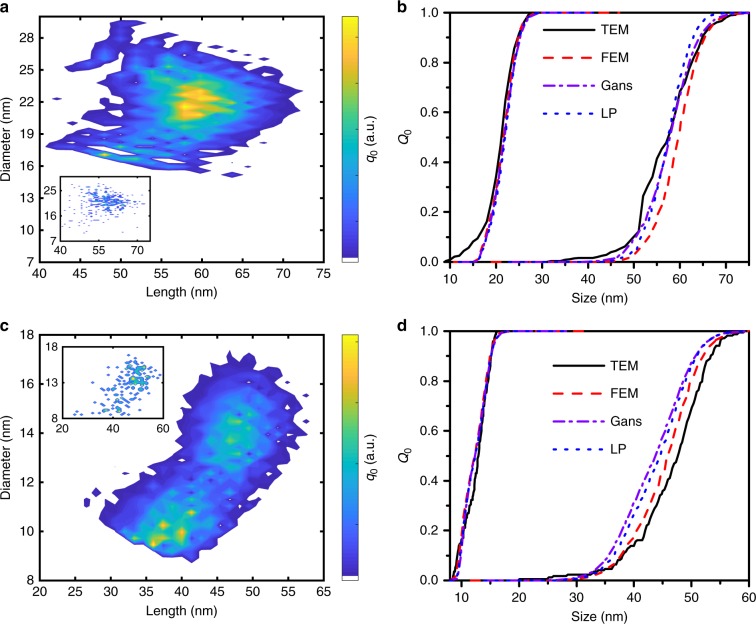
Resulting size distributions for the second and third CTAB sample. Comparison of **a** two-dimensional length and diameter distribution (Gans), **b** the corresponding two one-dimensional size distributions for CTAB sample 2 for the different optical models and **c** the two-dimensional size distribution (Gans), **d** the corresponding two one-dimensional size distributions for CTAB sample 3 with results from TEM analysis. The insets show the size distribution of nanorods obtained by TEM with **a** 320 and **c** 170 particles counted

The UV−Vis extinction spectra for a second CTAB sample were similarly extracted from 0.01 to 0.88 in the cumulative sedimentation coefficient distribution (see Supplementary Figure 27), while the third CTAB sample was analyzed from 0.01 to 0.60 (see Supplementary Figure 29). The results are depicted in Fig. 6 using 1D and 2D distributions for the second and third CTAB sample. Additional distributions (LP, FEM, TEM) are shown in Supplementary Figures 28, 30 and 31. In Fig. 6c, a clear correlation between length and diameter of the nanorods as well as two maxima is observable. The information obtained by our multidimensional approach enables thus to determine absolute size values of gold nanorods in one single experiment.

Often, syntheses produce distributions of nanoparticles with different shapes as it is the case for the third CTAB sample, which contains notable amounts of spheres. Therefore, higher sedimentation coefficients of the third CTAB sample were not taken into account for further analysis (see Supplementary Figures 41 and 29). It must be noted that the selected parts of the cumulative sedimentation coefficient distribution were chosen according to the shape of the distribution and the underlying optical signal, utilizing the different optical footprints of spherical nanoparticles compared to nanorods. Flat regions for high sedimentation coefficients are excluded as mostly agglomerates or other non-rod-shaped nanoparticles are represented there. These nanoparticles can only be detected for a very short time at the beginning of the experiment with all other nanoparticles being present in the background. Analyzing these nanoparticles results in altering the mean sedimentation coefficient and may change the optical spectrum of the overall sample containing rods and spheres. The extinction spectra obtained from the MWL-AUC allow discarding these parts of the sedimentation coefficient distribution from further analysis due to the unique optical fingerprint of the gold nanospheres (see Supplementary Figure 32). Additional analysis for the gold nanospheres with the fitted ${\it{\epsilon }}_{\mathrm{m}},h$ values (Gans) allows even deducing the ratio of the number of concentrations of spheres in the high sedimentation coefficient fraction to the number of rods being analyzed. For the third CTAB sample, we obtained a value of 3.7% for this fraction. The respective equations, spectra and size distributions can be found in Supplementary Note 6 and Supplementary Figure 32. The analysis of spheres having similar sedimentation properties than the rods will be subject of future work. Remarkably, the analysis of the rods is not affected by the presence of spheres, as the results from TEM analysis and AUC measurements are found to match very well (see Fig. 6).

**Fig. 7:**
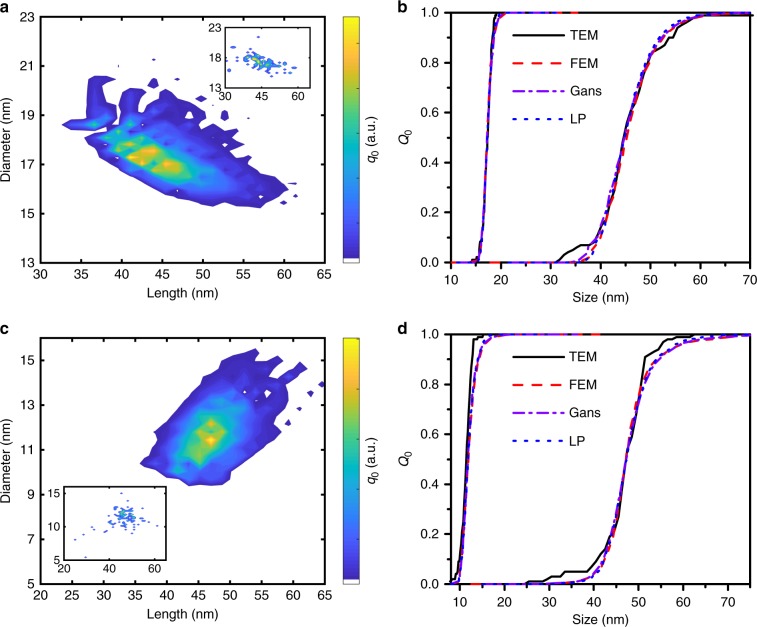
Resulting size distributions for the first and second citrate sample. Comparison of **a** 2D length and diameter distribution (Gans), **b** the corresponding two one-dimensional size distributions for citrate sample 1 for the different optical models and **c** the two-dimensional size distribution (Gans), **d** the corresponding two one-dimensional size distributions for citrate sample 2 in comparison with results from TEM analysis. The insets show the size distribution of nanorods obtained by TEM. One hundred particles were TEM-counted for each sample

To further prove the applicability of the proposed method, two samples with shells consisting of citrate were analyzed. The best results for citrate sample 1 in the interval 0.02−0.9 (Supplementary Figure 33) of the cumulative sedimentation coefficient distribution at 480 nm were obtained for the parameters given in Table 1. The TEM and the AUC distributions are matching quite well (Fig. 7a and Supplementary Figures 34 and 35). The citrate sample 2 was analyzed in the cumulative sedimentation coefficient interval between 0.02 and 0.98 using the obtained values. The results of the 2D distributions and the 1D length and diameter distributions are shown in Fig. 7b as well. Additional information on the spectra and the analysis can be found in the Supplementary Information (Supplementary Figures 36 and 37).

## Discussion

As the validation by simulations showed, the proposed OBC method is able to determine 2D size distributions with great precision. The data clearly show that the results are very promising independent of the applied optical model. Nevertheless, some differences were found. While the two analytical models allow matching the cumulative size and shape distributions from TEM almost perfectly by adjustment of $\epsilon _{\mathrm{m}},h$ (Figs. 5 and 7), the optically more accurate FEM model cannot retrieve the distributions equally well. As mentioned beforehand, several parameters can influence the modeled spectra of gold nanorods (diameter, length, optical properties of bulk material, end cap geometry, rod geometry, size and structure of the ligand shell as well as its optical properties). Hence, the adjustment of all these parameters by comparison to a macroscopically measured extinction spectra is for the time being beyond reach, especially as polydispersity is inevitable.

**Fig. 8:**
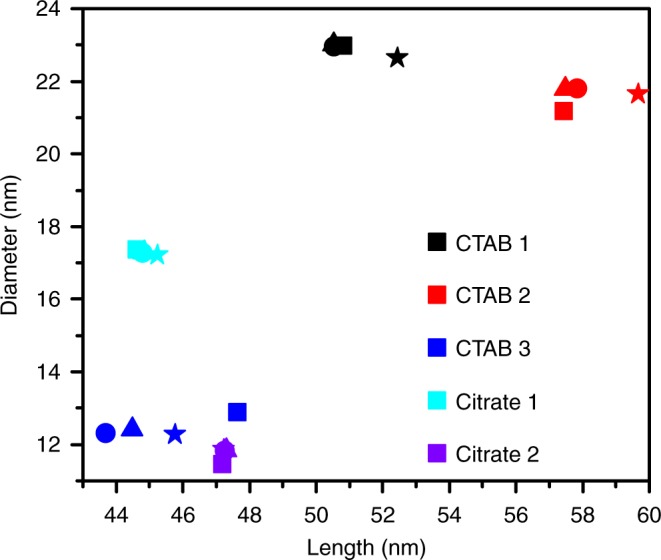
Median geometrical parameters deduced from MWL-AUC and TEM analysis. Samples are indicated by the given color, symbols correspond to the applied methods: (squares: TEM), (circles: Gans), (triangles: LP) and (stars: FEM)

Thus, an effective optical model representing at best the optical properties of the macroscopic sample must be employed. The concept of effective quantities is common for particle size analysis^23,55^, e.g. for the description of particle sizes of nonspherical particles via equivalent volumes. In the case of the two analytical models, this concept can be used by introducing an effective value of $\epsilon _{\mathrm{m}}$ with small numerical effort. The transfer of these effective optical models to other samples works quite well (see Fig. 8). Sample CTAB 3 shows the largest discrepancy with deviations smaller than 8.5%. The effective optical models allow slightly better size analysis than the FEM model. This is remarkable, since the Gans and the LP model are different regarding their physical foundation. It is thus preferable to use the effective optical models to analyze the gold nanorod samples. The FEM model is in principle the more accurate method, but its applicability is limited due to the partly unknown large and distributed parameter space and due to the huge computational effort. Despite these small inaccuracies in the optical modeling, the OBC method has the ability to determine full 2D size distributions and is expected to profit from future developments in optical modeling, allowing then to determine absolute concentrations of samples with even higher precision.

In terms of experimental accuracy, the resolution of the method depends also on the spectral resolution of the optical detector system. In our setup, hardware improvements achieving better resolution and sensitivity in the NIR will further enhance the performance of the method in the future. This is equally applicable for the size range of the analyzed gold nanorods. While smaller plasmonic particles than the ones involved in this study can be investigated using higher rotor speeds, the analysis of particles with optical features exceeding 1000 nm is limited up to now by our current instrumentation, but novel NIR setups are under development^56^. While TEM has a better accuracy for the analysis of individual particles, sample preparation is difficult and there is a lack of reproducibility due to the limited number of images taken for statistical analysis. The statistics of the OBC method is superior as all particles (typically billions of nanorods per ml) are subjected to sedimentation and contribute to the measurement signal. MWL-AUC offers higher throughput in comparison to conventional TEM-analysis, as three samples can typically be measured largely autonomously in parallel and fully analyzed within 4 h. A slight discrepancy can be found only for small particles which may be explained by the aggregation of the finest particles with larger rods. The coagulation of smaller particles with bigger particles is enhanced and aggregated particles can be analyzed by TEM but not by the OBC method. Therefore smaller particles that are part of larger aggregates get lost in the analysis. Boundary conditions of the new method are therefore stable dispersions and prior knowledge of particle shape. The OBC method can be further improved by the availability of better optical and hydrodynamic models.

In this study an OBC technique that combines sedimentation and optical data from an AUC equipped with multiwavelength detector is presented to measure the 2D length and diameter distributions of gold nanorods in one single experiment. Using simulation and experimental data, it is shown to be a powerful and accurate tool for the precise determination of the length and diameter distributions. Utilizing the different optical signatures of nanospheres and nanorods, this method can also be used to measure the relative concentrations of different species and allows one to estimate the thickness of different ligand shells on the particles. The methodology can be also tailored for other plasmonic nanoparticles, provided that the required information of the size, shape or composition can be obtained by analyzing the extracted spectra. We believe that this work will contribute to the fast and reliable characterization of plasmonic nanoparticles during synthesis and in numerous applications.

## Methods

### Materials

Gold nanorods from Alfa Aesar (46331, 46359, 46810) stabilized with CTAB in water were used for the experiments. These samples are referred to as CTAB samples 1, 2 and 3, respectively. The dimensions of the nanorods are based on the TEM analysis with 170−320 gold nanorods that depends on the sample and closely matches the data provided by the suppliers. The samples were diluted (2:1) with millipore water. Furthermore, two samples stabilized with citrate were bought from nanoComposix. These samples are referred to as citrate samples 1 and 2. The size distributions of the gold nanorods for the citrate samples were determined by the supplier company via TEM. Samples were diluted (5:1). All samples were exposed to an ultrasonic bath for 10 min. The volume fractions and the expected particle−particle distance in solution are given in Supplementary Note 8.

### Analytical ultracentrifugation

An Optima L-90K preparative ultracentrifuge from Beckman Coulter equipped with a multiwavelength detector (MWL-AUC) was used for dynamic rotor speed experiments in an An-60 Ti analytical rotor at 20 °C. Fast sedimentation of gold nanorods was achieved at a constant speed of 2000 rpm (CTAB sample one and two), 3000 rpm (citrate sample one) or 4000 rpm (CTAB sample 3 and citrate sample 2), allowing also to accumulate multiple lamp flashes on the spectrometer. Details of the optical setup as well as the data acquisition can be found in the literature^26,41^. Titanium centerpieces with an optical path length of 12 mm as well as sapphire windows from Nanolytics were utilized for all experiments. The data were collected at the radial position 6.9 cm with an Ocean Optics Flame-S-XR1-ES Spectrometer (200-1000 nm) for CTAB samples 1 and 2 and an Ocean Optics Flame-S Spectrometer (475-1100 nm) for CTAB sample 3 and citrate samples 1 and 2. The intensity data obtained during the run were transformed to extinction data after the run. Extinction sedimentation coefficient distributions were derived using a direct boundary model for the dynamic rotor speed experiment^57^. Spectral data from MWL-AUC experiments are given in Supplementary Figures 38-40.

### TEM

Conventional TEM analysis for CTAB samples 1, 2 and 3 was carried out with a Philipps CM30 TWIN/STEM (FEI Company) equipped with a LaB_6_ filament and operated at 300 kV accelerating voltage. Images were recorded in bright-field TEM mode using a charged coupled device (CCD) camera from TVIPS (Tietz Video and Processing Systems GmbH) with an image size of 1024 × 1024 pixels^2^. Samples were prepared on lacey carbon support films (Plano GmbH) by drop-casting of the diluted and ultrasonically treated CTAB samples 1, 2 and 3, respectively. It is noteworthy that the samples used for TEM analysis were identical to the samples used for the AUC experiments. Length and diameter distributions of the distinct gold nanorods samples were measured from the TEM images using ImageJ 1.47v.

### Calculations

All calculations were performed in MATLAB^©^ Version 2015b and 2018a.

### Simulations

The concentration distributions of nanoparticles were implemented using Gaussian distributions of adjustable mean and standard deviation for length and diameter of the bare nanorod on a grid of 500 × 500 data points. For each nanoparticle the sedimentation coefficients taking into account the effect of the CTAB ligand on the size and density and absorption spectra were calculated. The sedimentation coefficients were sorted and classified in intervals, allowing for summing up the corresponding optical spectra for each interval and setting up the same three-dimensional data space that can be derived from a dynamic rotor speed experiment.

Material parameters for the simulations and partly analysis were *ρ*_core_ = 19,300 kg m^−3^, used ligand densities were *ρ*_CTAB_ = 1458 kg m^−3^ calculated from ref. ^58^, *ρ*_Citrate_ = 1660 kg m^−3^, other parameters were *ρ*_solvent_ = 998.23 kg m^−3^, *η* = 1.002 mPas, *h* = 4 nm and ${\it{\epsilon }}_{\mathrm{m}} = 2$. The density of the shell was assumed to be the mean density of the solvent and the ligand.

### Finite element method (FEM)

The average extinction cross-section spectra of a randomly oriented nanorod defined as cylinder with spherical endcaps (Supplementary Figure 1) described by its total length *l* and diameter *d* is given by:

$$\sigma _{{\mathrm{ext}}}\left( {d,l;\lambda } \right) = \frac{1}{{2\pi }}\mathop {\int }\nolimits_0^{2\pi } \mathop {\int }\nolimits_0^\pi \mathop {\int }\nolimits_0^{2\pi } \hat \sigma _{{\mathrm{ext}}}\left( {d,l;\lambda ,P(\theta ,\phi ,\psi ),D\left( {\phi ,\psi } \right)} \right)\mathrm{sin}\left( \phi \right){\mathrm d}\theta {\mathrm d}\phi {\mathrm d}\psi$$

with *D*(*φ*,*θ*) as the direction of the incident light and *P*(*θ*, *φ*,* ψ*) as its polarization. In the following, we describe how *σ*_ext_ is obtained from computer simulations in the framework of which the time-harmonic Maxwell equation is solved by the FEM. In the core of the latter, tessellations are generated by TETGEN^59^ and Nedelec basis functions^60^ are applied. For the refractive index of gold we used data of Johnson and Christy^49^, for the refractive index of water we chose *n* = 1.33. The overall FEM is implemented in MATLAB R2015b. However, the part with highest computational complexity, i.e. the solution of the resulting complex valued linear system of equations, is implemented in C using HSL_MA86^61^ via the MATLAB-MEX interface. To be able to evaluate *σ*_ext_(*d*,* l*;* λ*) for any wavelength *λ* ∊ [350 nm,1350 nm] with a given error tolerance an adaptive wavelength discretization is applied to reduce the overall computation time. To describe the dependency of *σ*_ext_(*d*, *l*; *λ*) on *l* and *d*, *σ*_ext_(*d*, *l*; *λ*) is evaluated on a 2D nonuniform grid (maximal grid width 5 nm) and interpolated. The interpolation is performed in two steps: In the first step, the main peak of the spectrum is fitted by the following formula:

$$\sigma _{{\mathrm{ext}}}\left( {d,l;\lambda } \right) \approx \frac{{a\left( {d,l} \right)}}{{b\left( {d,l} \right)\left( {\lambda - c\left( {d,l} \right)} \right)^2 + 1}},\\ \sigma _{{\mathrm{ext}},{\mathrm{res}}}\left( {d,l;\lambda } \right) : = \sigma _{{\mathrm{ext}}}\left( {d,l;\lambda } \right) - \frac{{a\left( {d,l} \right)}}{{b\left( {d,l} \right)\left( {\lambda - c\left( {d,l} \right)} \right)^2 + 1}}.$$

Here, the coefficients *a*(*d*, *l*), *b*(*d*, *l*) and *c*(*d*, *l*) are fitted for every value *d* and *l* on the grid. Then the coefficients and the residual spectra *σ*_ext,res_(*d*,* l*;* λ*) are interpolated. This results in a very accurate interpolation of the spectrum for all *l* and *d* in the desired region which is in sharp contrast to classical linear interpolation of spectra (please see Supplementary Note 9 for further information).

### Code availability

The codes used for this study are available from the corresponding author upon request.

## Electronic supplementary material

Supplementary Information

## Data Availability

The data used for this study are available from the corresponding author upon request.

## References

[1] Sun Y, Tang Z (2017). Plasmonic particles—now tailored to your needs. Part. Part. Syst. Charact..

[2] Olson J (2015). Optical characterization of single plasmonic nanoparticles. Chem. Soc. Rev..

[3] Gans R (1912). Über die Form ultramikroskopischer Goldteilchen. Ann. Phys..

[4] Chen H, Shao L, Li Q, Wang J (2013). Gold nanorods and their plasmonic properties. Chem. Soc. Rev..

[5] Dreaden EC, Alkilany AM, Huang X, Murphy CJ, El-Sayed MA (2012). The golden age: gold nanoparticles for biomedicine. Chem. Soc. Rev..

[6] Mackey MA, Ali MRK, Austin LA, Near RD, El-Sayed MA (2014). The most effective gold nanorod size for plasmonic photothermal therapy: theory and in vitro experiments. J. Phys. Chem. B.

[7] Zhang Z (2014). Near infrared laser-induced targeted cancer therapy using thermoresponsive polymer encapsulated gold nanorods. J. Am. Chem. Soc..

[8] Jain PK, Huang X, El-Sayed IH, El-Sayed MA (2007). Review of some interesting surface plasmon resonance-enhanced properties of noble metal nanoparticles and their applications to biosystems. Plasmonics.

[9] La Zerda Ade (2015). Optical coherence contrast imaging using gold nanorods in living mice eyes. Clin. Exp. Ophthalmol..

[10] Cao J, Sun T, Grattan KT (2014). Gold nanorod-based localized surface plasmon resonance biosensors. A review. Sens. Actuator B Chem..

[11] Huang H (2013). Multiplex plasmonic sensor for detection of different metal ions based on a single type of gold nanorod. Anal. Chem..

[12] Solowan HP, Kryschi C (2017). Facile design of a plasmonic nanolaser. Condens. Matter.

[13] Ye X, Zheng C, Chen J, Gao Y, Murray CB (2013). Using binary surfactant mixtures to simultaneously improve the dimensional tunability and monodispersity in the seeded growth of gold nanorods. Nano Lett..

[14] Lohse SE, Murphy CJ (2013). The quest for shape control. A history of gold nanorod synthesis. Chem. Mater..

[15] Tatini F (2014). Size dependent biological profiles of PEGylated gold nanorods. J. Mater. Chem. B.

[16] Liu H, Pierre-Pierre N, Huo Q (2012). Dynamic light scattering for gold nanorod size characterization and study of nanorod–protein interactions. Gold. Bull..

[17] Glidden M, Muschol M (2012). Characterizing gold nanorods in solution using depolarized dynamic light scattering. J. Phys. Chem. C.

[18] Hubert F (2012). Growth and overgrowth of concentrated gold nanorods. Time resolved SAXS and XANES. Cryst. Growth Des..

[19] Schindler T, Walter J, Peukert W, Segets D, Unruh T (2015). In situ study on the evolution of multimodal particle size distributions of ZnO quantum dots: some general rules for the occurrence of multimodalities. J. Phys. Chem. B.

[20] Schindler T (2015). A combined SAXS/SANS study for the in situ characterization of ligand shells on small nanoparticles: the case of ZnO. Langmuir.

[21] Thajudeen T, Walter J, Srikantharajah R, Lübbert C, Peukert W (2017). Determination of the length and diameter of nanorods by a combination of analytical ultracentrifugation and scanning mobility particle sizer. Nanoscale Horiz..

[22] Elazzouzi-Hafraoui S (2008). The shape and size distribution of crystalline nanoparticles prepared by acid hydrolysis of native cellulose. Biomacromolecules.

[23] Rawat VK (2016). Two dimensional size–mass distribution function inversion from differential mobility analyzer–aerosol particle mass analyzer (DMA–APM) measurements. J. Aerosol Sci..

[24] Walter J (2016). 2D analysis of polydisperse core-shell nanoparticles using analytical ultracentrifugation. Anal. (Lond.)..

[25] Demeler B (2014). Characterization of size, anisotropy, and density heterogeneity of nanoparticles by sedimentation velocity. Anal. Chem..

[26] Walter J, Peukert W (2016). Dynamic range multiwavelength particle characterization using analytical ultracentrifugation. Nanoscale.

[27] Walter J (2015). Simultaneous analysis of hydrodynamic and optical properties using analytical ultracentrifugation equipped with multiwavelength detection. Anal. Chem..

[28] Link S, Mohamed MB, El-Sayed MA (1999). Simulation of the optical absorption spectra of gold nanorods as a function of their aspect ratio and the effect of the medium dielectric constant. J. Phys. Chem. B.

[29] Eustis S, El-Sayed MA (2006). Determination of the aspect ratio statistical distribution of gold nanorods in solution from a theoretical fit of the observed inhomogeneously broadened longitudinal plasmon resonance absorption spectrum. J. Appl. Phys..

[30] Khlebtsov NG (2008). Determination of size and concentration of gold nanoparticles from extinction spectra. Anal. Chem..

[31] Near RD, Hayden SC, Hunter RE, Thackston D, El-Sayed MA (2013). Rapid and efficient prediction of optical extinction coefficients for gold nanospheres and gold nanorods. J. Phys. Chem. C.

[32] Pecharromán C, Pérez-Juste J, Mata-Osoro G, Liz-Marzán LM, Mulvaney P (2008). Redshift of surface plasmon modes of small gold rods due to their atomic roughness and end-cap geometry. Phys. Rev. B.

[33] Hu ZJ (2014). Fast characterization of gold nanorods ensemble by correlating its structure with optical extinction spectral features. AIP Adv..

[34] Carbó-Argibay E (2010). The crystalline structure of gold nanorods revisited: evidence for higher-index lateral facets. Angew. Chem. Int. Ed..

[35] Prescott SW, Mulvaney P (2006). Gold nanorod extinction spectra. J. Appl. Phys..

[36] Ungureanu C, Rayavarapu RG, Manohar S, van Leeuwen TG (2009). Discrete dipole approximation simulations of gold nanorod optical properties. Choice of input parameters and comparison with experiment.. J. Appl. Phys..

[37] Goris B (2012). Atomic-scale determination of surface facets in gold nanorods. Nat. Mater..

[38] Park K (2013). Growth mechanism of gold nanorods. Chem. Mater..

[39] Schuck, P., Zhao, H. & Brautigam, C. A. *Basic Principles of Analytical Ultracentrifugation* (CRC Press; MyiLibrary, Boca Raton, La Vergne, 2015).

[40] Uchiyama Susumu, Arisaka Fumio, Stafford Walter F., Laue Tom (2016). Analytical Ultracentrifugation.

[41] Walter J (2014). Multidimensional analysis of nanoparticles with highly disperse properties using multiwavelength analytical ultracentrifugation. ACS Nano.

[42] Strauss HM (2008). Performance of a fast fiber based UV/Vis multiwavelength detector for the analytical ultracentrifuge. Colloid Polym. Sci..

[43] Bhattacharyya, S. K. et al. Development of a fast fiber based UV-Vis multiwavelength detector for an ultracentrifuge. In *Analytical Ultracentrifugation VIII. Progress in Colloid and Polymer Science* Vol. 131 (eds Wandrey C. & Cölfen H.) 9–22 (Springer, Berlin, Heidelberg, 2006).

[44] Karabudak E (2016). Simultane Bestimmung spektraler Eigenschaften und Größen von multiplen Partikeln in Lösung mit Subnanometer-Auflösung. Angew. Chem..

[45] Zhang J (2017). Spectral and hydrodynamic analysis of West Nile Virus RNA−protein interactions by multiwavelength sedimentation velocity in the analytical ultracentrifuge. Anal. Chem..

[46] Hansen S (2004). Translational friction coefficients for cylinders of arbitrary axial ratios estimated by Monte Carlo simulation. J. Chem. Phys..

[47] Ortega A, García de la Torre J (2003). Hydrodynamic properties of rodlike and disklike particles in dilute solution. J. Chem. Phys..

[48] Yu R, Liz-Marzán LM, de Abajo G, Javier F (2017). Universal analytical modeling of plasmonic nanoparticles. Chem. Soc. Rev..

[49] Johnson PB, Christy RW (1972). Optical constants of the noble metals. Phys. Rev. B.

[50] Lagarias JC, Reeds JA, Wright MH, Wright PE (1998). Convergence properties of the Nelder−Mead simplex method in low dimensions. SIAM J. Optim..

[51] Walter J (2016). 2D analysis of polydisperse core-shell nanoparticles using analytical ultracentrifugation. Anal. (Lond.)..

[52] Sadovnikov SI, Gusev AI, Gerasimov EY, Rempel AA (2015). Facile synthesis of Ag2S nanoparticles functionalized by carbon-containing citrate shell. Chem. Phys. Lett..

[53] Gómez-Graña S (2012). Surfactant (bi)layers on gold nanorods. Langmuir.

[54] da Silva JA, Meneghetti MR (2018). New aspects of the gold nanorod formation mechanism via seed-mediated methods revealed by molecular dynamics simulations. Langmuir.

[55] Wawra Simon E., Thoma Martin, Walter Johannes, Lübbert Christian, Thajudeen Thaseem, Damm Cornelia, Peukert Wolfgang (2018). Ionomer and protein size analysis by analytical ultracentrifugation and electrospray scanning mobility particle sizer. European Biophysics Journal.

[56] Pearson Joseph, Cölfen Helmut (2018). LED based near infrared spectral acquisition for multiwavelength analytical ultracentrifugation: A case study with gold nanoparticles. Analytica Chimica Acta.

[57] Schuck P, Rossmanith P (2000). Determination of the sedimentation coefficient distribution by least-squares boundary modeling. Biopolymers.

[58] Bhattarai, A., Chatterjee, S. K. & Jha, K. Density and partial molar volume of cetyltrimethylammonium bromide in the presence and absence of KCl and NaCl in aqueous media at room temperature. *Phys. Chem.* **5**, 1–5 (2015).

[59] Si H (2015). TetGen, a Delaunay-based quality tetrahedral mesh generator. ACM Trans. Math. Softw..

[60] Schöberl J, Zaglmayr S (2005). High order Nedelec elements with local complete sequence properties. COMPEL—Int. J. Comput. Math. Electr. Electron. Eng..

[61] HSL & Science & Technology Facilities Council. A collection of Fortran codes for large scale scientific computation. http://www.hsl.rl.ac.uk (2013).

