# Supplementary material for: Determination of the two-dimensional distributions of gold nanorods by multiwavelength analytical ultracentrifugation
Source: Nat Commun. 2018 Nov 21;9:4898. doi: 10.1038/s41467-018-07366-9 (PMC6249260; doi:10.1038/s41467-018-07366-9)
Supplement: Supplementary file 1 — Supplementary Information [file 41467_2018_7366_MOESM1_ESM.pdf]

Supplementary Information

Determination of the Two-Dimensional Distributions of Gold Nanorods by Multiwavelength Analytical Ultracentrifugation

Simon E. Wawra, Lukas Pflug, Thaseem Thajudeen, Carola Kryschi, Michael Stingl, Wolfgang Peukert

Correspondence and requests for materials should be addressed to W.P. (email: wolfgang.peukert@fau.de).

Supplementary Note 1: Hydrodynamic description

As being noted in the main manuscript, m, ρ_P, x_V can be described as a function of the aspect ratio $p = \frac{l}{d}$. The geometry of a gold nanorod is depicted in Supplementary Figure 1.

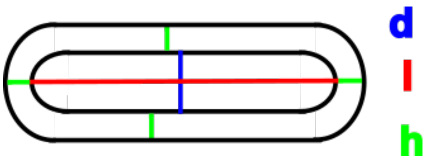

Supplementary Figure 1 Geometrical parameters used for the hydrodynamic description of the gold nanorod. For reasons of clarity, the rotational symmetry in terms of the axis l is not shown.

Since the gold nanorods exhibit hemispherical end caps (see Supplementary Figure 41), this needs to be taken into account due to the high bulk density of gold, especially for small aspect ratio systems. Thus, the volume of the nanorod V can be calculated according to Supplementary Equation 1.

$$V = (l - d) \cdot \frac{d^2}{4}\pi + \frac{\pi d^3}{6} = (pd - d) \cdot \frac{d^2}{4}\pi + \frac{\pi d^3}{6} = d^3\pi \cdot \left(\frac{p}{4} - \frac{1}{12}\right) \tag{1}$$

Length and diameter of the stabilized particle l_s and d_s can be obtained by adding the height of the ligand shell h:

$$l_s = l + 2 \cdot h \tag{2}$$

$$d_s = d + 2 \cdot h \tag{3}$$

This value allows calculating the aspect ratio of the stabilized particle p_s which is required for the calculation of the frictional ratio:

$$p_s = \frac{l_s}{d_s} = \frac{l + 2 \cdot h}{d + 2 \cdot h} = \frac{pd + 2 \cdot h}{d + 2 \cdot h} \tag{4}$$

Supplementary Equation 5 gives then the volume of the stabilized particle.

$$V_s = (d + 2 \cdot h)^3\pi \cdot \left(\frac{p_s}{4} - \frac{1}{12}\right) \tag{5}$$

Furthermore, the stabilized particle density ρ_P is obtained from the density of the ligand shell ρ_{shell} and the density of the bare rod ρ_{core}.

$$\rho_P = \frac{m_{rod} + m_{shell}}{V_s} = \frac{V_s\rho_{shell} + V(\rho_{core} - \rho_{shell})}{V_s} \tag{6}$$

The density of the shell is assumed to be the mean value of solvent density and ligand density:

$$\rho_{\text{shell}} = \frac{(\rho_{\text{ligand}} + \rho_{\text{solvent}})}{2} \tag{7}$$

Finally the volume equivalent diameter x_V has to be calculated:

$$x_V = \left(\frac{6}{\pi} V_s\right)^{\frac{1}{3}} \tag{8}$$

With these equations, the hydrodynamic properties of the stabilized gold nanorod can be described using the geometry of the bare nanorod and material parameters.

The surface of a rod is determined according to:

$$A = (l - d) \cdot d\pi + \pi d^2 = d\pi(pd - d) + \pi d^2 = d^2\pi(p - 1) + \pi d^2 = \pi d^2 p \tag{9}$$

Supplementary Note 2: Gans Model

Supplementary Equations 10 and 11 define the the absorption α_{nkf} and the scattering cross-section σ_{nkf} of an ellipsoidal particle as a function of the shape factors $P_{J,nk}$. These can be obtained by using Supplementary Equation 12a and Supplementary Equation 12b for J=A, B, C as a function of the variable κ_k as defined in Supplementary Equation 12c.[1–3]

$$\alpha_{nkf} = \frac{2\pi V_{nk} \epsilon_m^{\frac{3}{2}}}{3\lambda_f} \sum_J \frac{\left(\frac{1}{P_{J,nk}^2}\right) \epsilon_{2f}}{\left\{\epsilon_{1f} + \left[\frac{1 - P_{J,nk}}{P_{J,nk}}\right] \epsilon_m\right\}^2 + \epsilon_{2f}^2} \tag{10}$$

$$\sigma_{nkf} = \frac{8\pi^3 V_{nk}^2 \epsilon_m^2}{9\lambda_f^4} \sum_J \frac{\left(\frac{1}{P_{J,nk}^2}\right) \cdot \left[(\epsilon_{1f} - \epsilon_m)^2 + \epsilon_{2f}^2\right]}{\left\{\epsilon_{1f} + \left[\frac{1 - P_{J,nk}}{P_{J,nk}}\right] \epsilon_m\right\}^2 + \epsilon_{2f}^2} \tag{11}$$

$$P_{A,nk} = P_{A,k} = \frac{1 - \kappa_k^2}{\kappa_k^2} \left[\frac{1}{2\kappa_k} \ln\left(\frac{1 + \kappa_k}{1 - \kappa_k}\right) - 1\right] \tag{12a}$$

$$P_{B,nk} = P_{B,k} = P_{C,k} = \frac{1 - P_{A,k}}{2} \tag{12b}$$

$$\kappa_k = \sqrt{1 - \left(\frac{1}{p_k}\right)^2} \tag{12c}$$

V_{nk} is the particle volume, ϵ_m is the dielectric constant of the surrounding medium and $\epsilon(\lambda_f) = \epsilon_{1f} + i\epsilon_{2f}$ is the complex dielectric constant of gold. Therefore, α_{nkf} denotes the absorption cross-section at wavelength λ_f of a particle within the nth extracted spectra/ sedimentation coefficient interval and having the aspect ratio p_k.

Since the absorption and scattering cross-section depend on the volume V_{nk} of the particle, the diameter d_{nk} is calculated for each aspect ratio p_k using the mean sedimentation coefficient s_n of the respective interval based on Equation 1 in the main manuscript.

Supplementary Note 3: Simulations

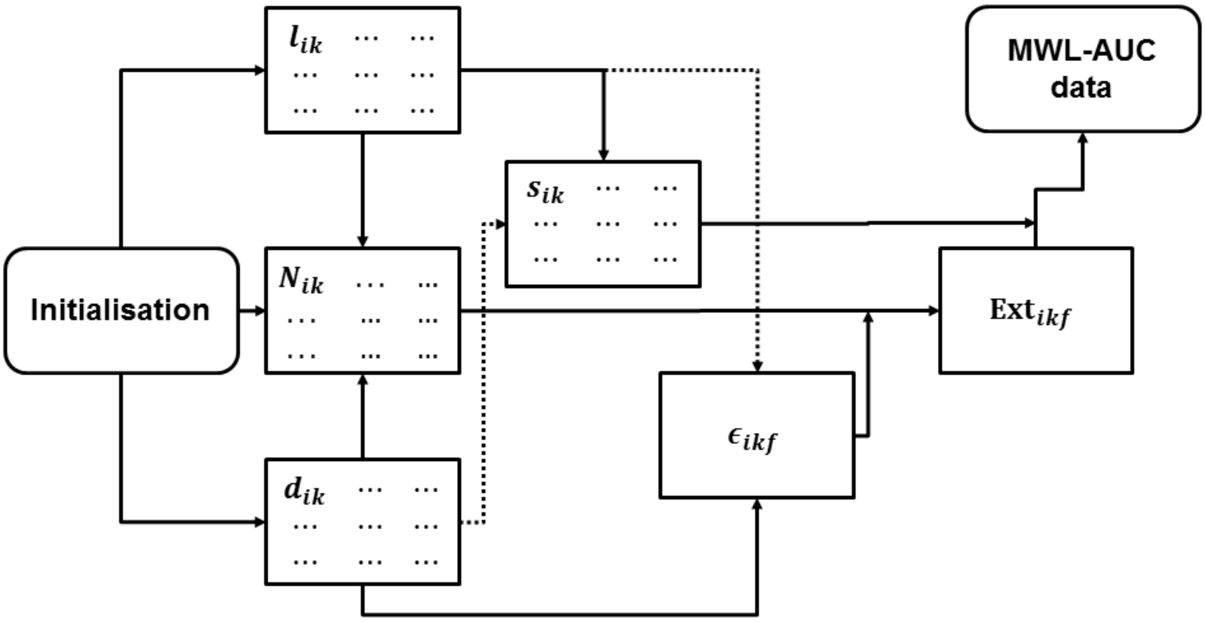

Supplementary Figure 2 Flow chart indicating the flow of information during the simulation of sedimenting gold nanorods.

For the simulation, the optical and the sedimentation properties for 500x500 nanorods with different number concentrations were calculated in order to mimic results from an experiment with MWL-AUC. The number concentration of each particle being identified by its length l_{ik} and diameter d_{ik} is calculated by means of a Gaussian distribution given in Supplementary Equation 13 with standard deviations σ and mean values μ.

$$\mathrm{N}_{ik} = \frac{1}{\sigma_l\sqrt{2\pi}} \cdot \frac{1}{\sigma_d\sqrt{2\pi}} \cdot \exp\left(-\frac{(d_{ik}-\mu_d)^2}{2\sigma_d^2}\right) \cdot \exp\left(-\frac{(l_{ik}-\mu_l)^2}{2\sigma_l^2}\right) \tag{13}$$

Therefore the ranges for l_{ik} and d_{ik} are chosen to be $\mu \pm 3\sigma$. In order to initialise all possible combinations of length and diameter one of the two parameters is linearly spaced along k the other along i. From l_{ik} and d_{ik} the sedimentation coefficient s_{ik} can be calculated for each particle by using Equation 1, Supplementary Equations 1-8 and the parameters given in the main manuscript.

Simultaneously, from the geometry of the gold nanorod, the extinction cross sections ϵ_{ikf} can be calculated for every wavelength λ_f . Together with the number density, the extinction is then calculated.

In order to mimic the data from an MWL-AUC experiment, the sedimentation coefficients are arranged in ascending order, while the corresponding spectra are added also in ascending order to arrive at the non-normalized cumulative sedimentation coefficient distribution for every wavelength.

The initial length and diameter distribution of the simulation is depicted in Supplementary Figure 3. For all models Supplementary Equation 13 was used to initiate the simulation. Regardless of the optical model, the input distributions can be well reproduced

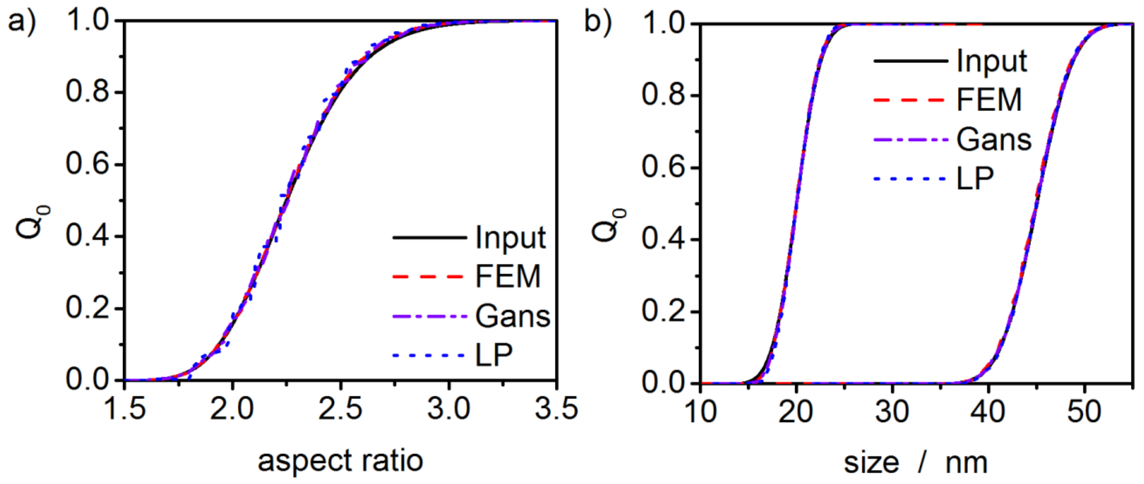

Supplementary Figure 3 Resulting one-dimensional size distributions for the simulations. Comparison of a) aspect ratio, b) length and diameter input and output distributions of the simulated data with prior knowledge of the relevant parameters. For every model, corresponding sets of sedimentation data were calculated and analysed.

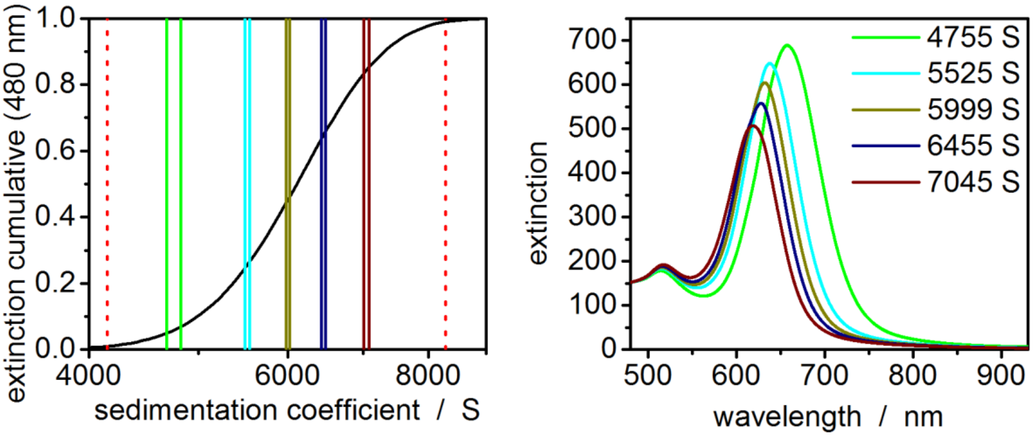

Supplementary Figure 4 Range of analysis (red) and selected sedimentation coefficient intervals and corresponding extracted spectra with mean sedimentation coefficients for the simulated data using the FEM model. For reasons of clarity not every spectra or interval is shown. The color of the intervals corresponds to the upper and lower boundary as well as to the extracted spectra. The interval length varies according to the cumulative distribution from 40 to 400 S.

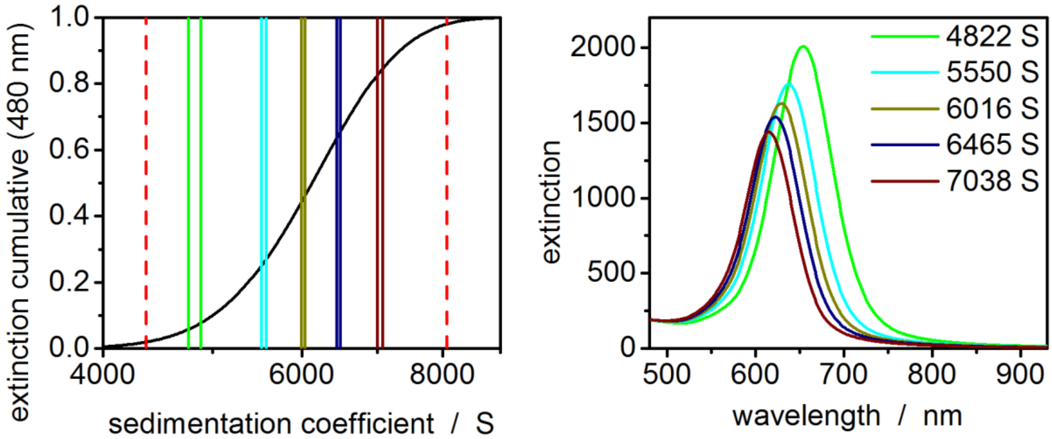

Supplementary Figure 5 Range of analysis (red) and selected sedimentation coefficient intervals and corresponding extracted spectra with mean sedimentation coefficients for the simulated data using the LP model. For reasons of clarity not every spectra or interval is shown. The color of the intervals corresponds to the upper and lower boundary as well as to the extracted spectra. The interval length varies according to the cumulative distribution from 40 to 400 S.

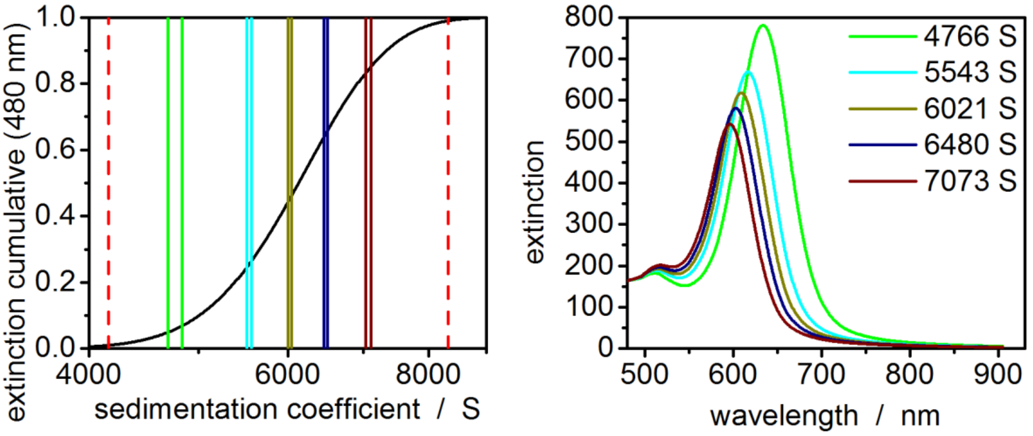

Supplementary Figure 6 Range of analysis (red) and selected sedimentation coefficient intervals and corresponding extracted spectra with mean sedimentation coefficients for the simulated data using the Gans model. For reasons of clarity not every spectra or interval is shown. The color of the intervals corresponds to the upper and lower boundary as well as to the extracted spectra. The interval length varies according to the cumulative distribution from 40 to 400 S.

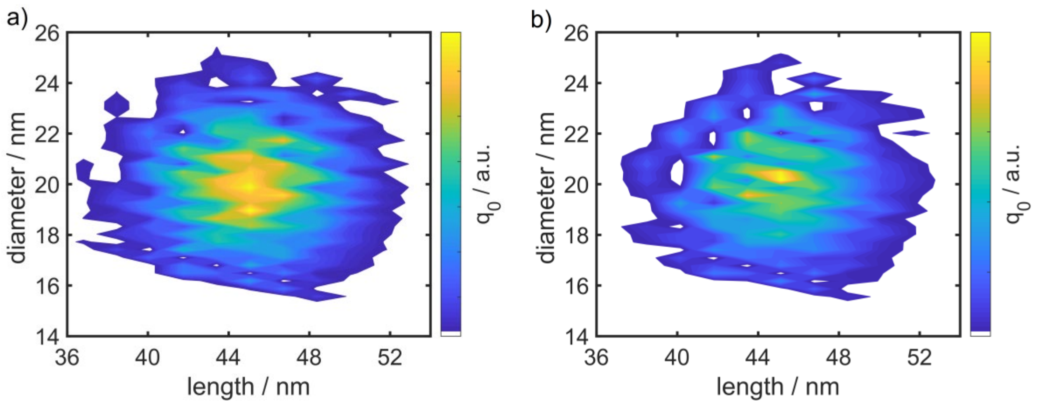

Supplementary Figure 7 Resulting two dimensional distributions for the simulations. a) shows Results for Gans and b) for the LP model.

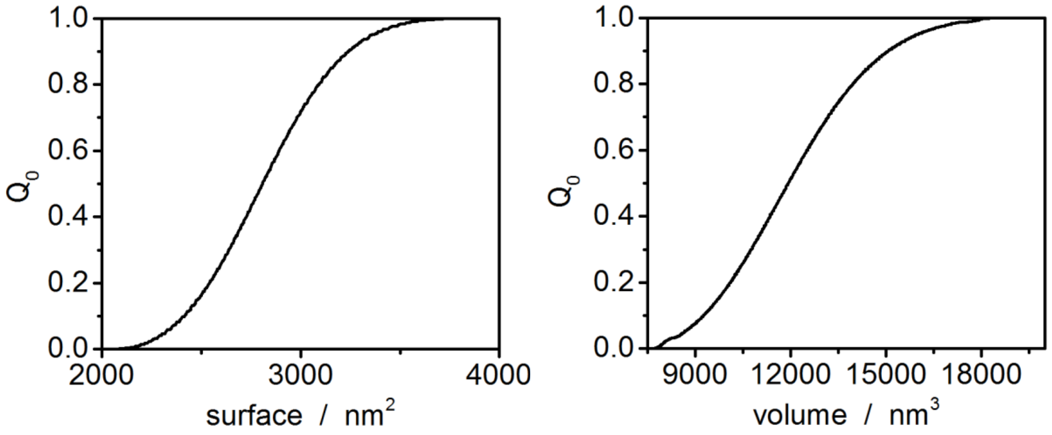

Supplementary Figure 8 Resulting cumulative surface and volume distribution of the fitted simulated data for the FEM model.

Supplementary Note 4: Effect of the selected intervals on the analysis

Supplementary Figure 9 shows the effect of the number of sedimentation coefficient intervals on the resulting length, diameter and aspect ratio distribution. Using only one interval causes a gradual increase of the aspect ratio and a steep increase of the diameter distribution. This is due to the fact that only a small number of species is fitted during the deconvolution of the spectra and that only one mean value of the sedimentation coefficient is used. The first issue could be resolved either by using regularization or by increasing the number of intervals, while the second issue will be resolved by increasing the resolution in the sedimentation coefficient, resulting in more intervals. Therefore, it can be ascertained that ten to fifty

sedimentation coefficient distributions should be enough to generate sufficiently smooth distribution curves.

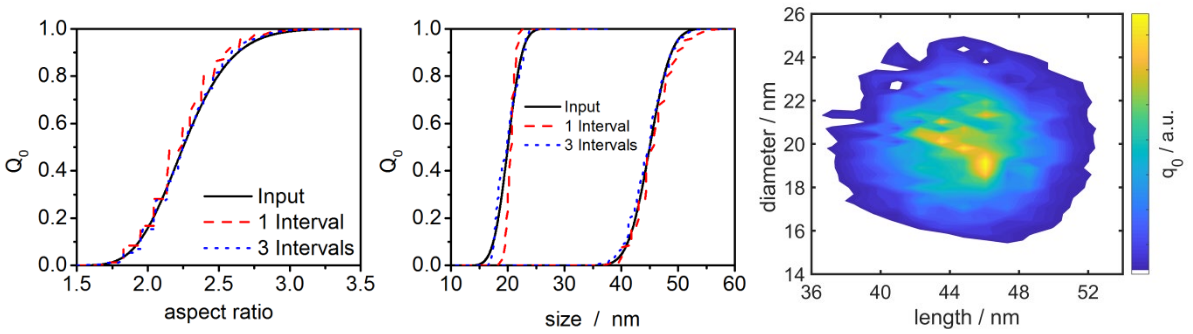

Supplementary Figure 9 Resulting diameter, length and aspect ratio distributions for different number of intervals as well as the 2D size distribution using 200 intervals for the FEM model.

Setting the sedimentation coefficient and using corresponding mean sedimentation coefficients contributes to an error in the retrieved diameter and length of the gold nanorod. However, calculations on a model gold nanorod (see Supplementary Figure 10) show that the relative error of the sedimentation coefficient is roughly two times bigger than the error of the final diameter. This reduction of error is also valid for the length as it can be directly calculated from the diameter and aspect ratio.

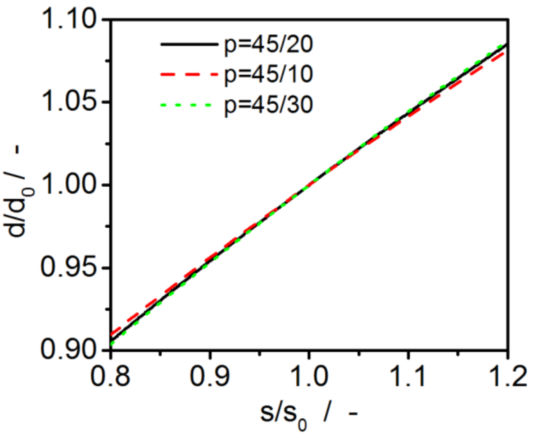

Supplementary Figure 10 Influence of deviating sedimentation coefficient on the resulting diameter. The length of the Au nanorod used for calculation was 45 nm, the used diameters are given in the figure. The aspect ratio was assumed to be accurately determined.

Supplementary Note 5: Aspect ratio distribution and solution space

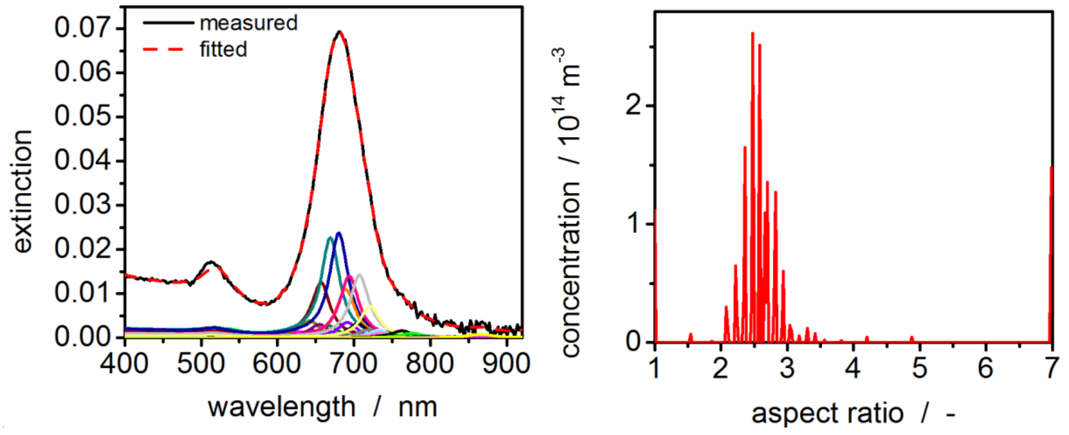

Supplementary Figure 11 Typical spectral analysis of the third sedimentation coefficient interval (5460 S) of the first CTAB sample and resulting concentration histogram using the Gans model. Please note that the species with lowest and highest aspect ratio are discarded as the corresponding nanorod spectra do not fit into the specified wavelength interval as specified in the main text.

As there are no restrictions implemented in the spectral fitting algorithm, the whole spectra range can be covered including species with very small and very high aspect ratio. Please note that the species with lowest and highest aspect ratio are discarded as the corresponding nanorod spectra do not fit into the specified wavelength interval as specified in the main text. In future work the obtained histogram may be modified by either smoothing or by regularization.

By separating the cumulative sedimentation coefficient distribution and fitting an aspect ratio distribution to the spectra of the respective interval, one confines the possible solutions of the overall 2D problem to a defined space. The solution space for each interval is shown in Supplementary Figure 12. Each line represents one interval having a certain sedimentation coefficient. Following the line means changing the aspect ratio, leading to changes in l and d at the same time.

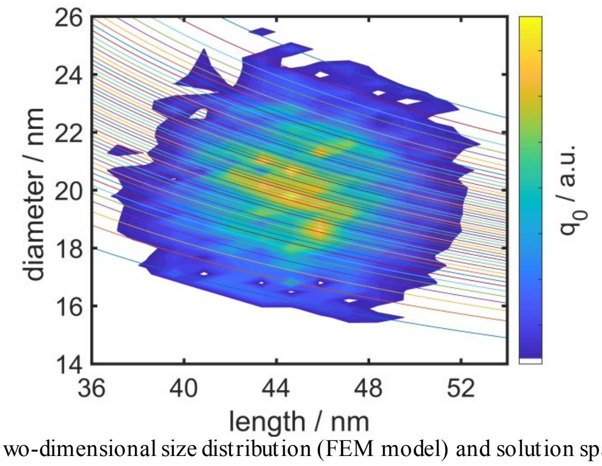

Supplementary Figure 12 Two-dimensional size distribution (FEM model) and solution space that is created by separating the simulated cumulative sedimentation coefficient distribution in 50 intervals. The different points on a iso-sved curve (species having the same sedimentation coefficient) correspond to different aspect ratios.

Supplementary Note 6: Analysis of gold nanospheres

The scattering and absorption cross sections of spherical nanoparticles with diameters much smaller than the wavelength, are given in Supplementary Equation 14 and 15.[1]

$$\alpha = \frac{18\pi V \epsilon_{\mathrm{m}}^{\frac{3}{2}}}{\lambda} \frac{\epsilon_2}{\{\epsilon_1 + 2 * \epsilon_{\mathrm{m}}\}^2 + \epsilon_2^2} \tag{14}$$

$$\sigma = \frac{24\pi^3 V^2 \epsilon_{\mathrm{m}}^2}{\lambda^4} \frac{(\epsilon_1 - \epsilon_{\mathrm{m}})^2 + \epsilon_2^2}{\{\epsilon_1 + 2 * \epsilon_{\mathrm{m}}\}^2 + \epsilon_2^2} \tag{15}$$

The diameter of the stabilized gold nanosphere d_{s} can be calculated according to Supplementary Equation 16. The density of the stabilized particle is given accordingly in Supplementary Equation 17.

$$d_{\mathrm{s}} = d + 2 \cdot h \tag{16}$$

$$\rho_{\mathrm{P}} = \frac{m_{\mathrm{sphere}} + m_{\mathrm{shell}}}{V_{\mathrm{sphere}}} = \frac{V_{\mathrm{s}}\rho_{\mathrm{shell}} + V(\rho_{\mathrm{core}} - \rho_{\mathrm{shell}})}{V_{\mathrm{s}}} \tag{17}$$

The sedimentation coefficient gives thus directly access to the diameter d of the gold nanorod:

$$s(d) = \frac{(d_{\mathrm{s}}(d))^2 \cdot (\rho_{\mathrm{P}}(d) - \rho_{\mathrm{s}})}{18\eta} \tag{18}$$

Supplementary Note 7: Stability of OBC-method against noise (FEM)

Due to the separation in sedimentation coefficient intervals, the used sedimentation coefficient is rather stable against experimental random noise. The deconvolution of the spectra, however, could be affected. In order to investigate this issue, Gaussian noise at different signal-to-noise-ratios was added to the (FEM) spectra of every interval.

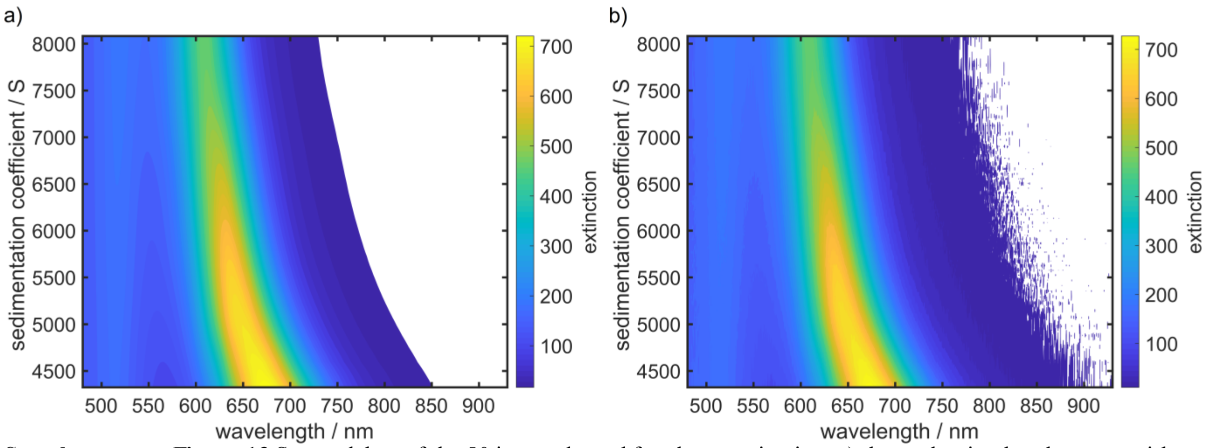

Supplementary Figure 13 Spectral data of the 50 intervals used for characterization. a) shows the simulated spectra without, while b) shows the spectra with added noise (signal-to-noise-ratio of 40dB). Please note that the given turbidity is based on the natural logarithm.

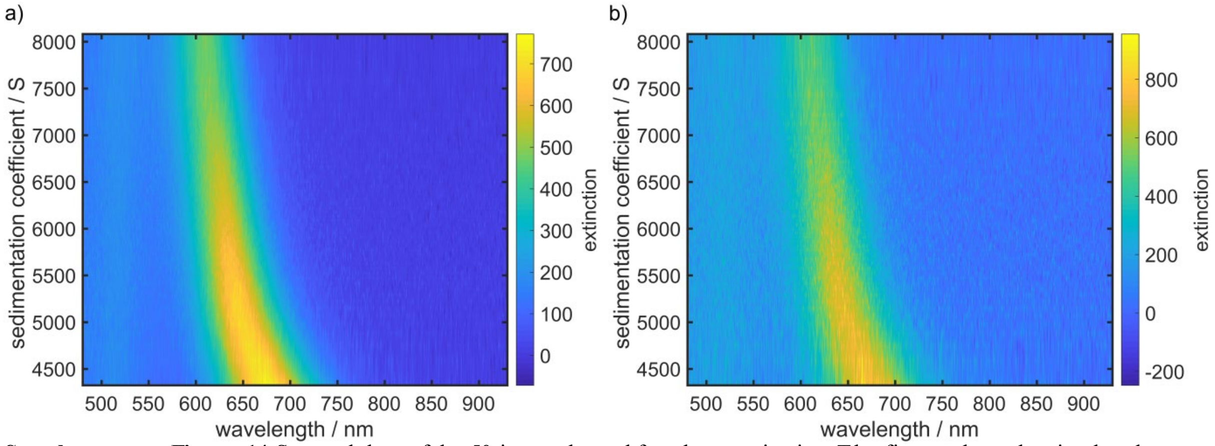

Supplementary Figure 14 Spectral data of the 50 intervals used for characterization. The figures show the simulated spectra with added noise, giving a signal-to-noise-ratio of a) 20 dB and b) 10 dB. Please note that the given turbidity is based on the natural logarithm.

The respective spectra are depicted in Supplementary Figure 13 and Supplementary Figure 14. The resulting two-dimensional size distributions are shown in Supplementary Figure 15 and Supplementary Figure 16. The results are very similar. Even though the shape of the distributions gets distorted to some extent, the results still provide information of excellent quality, especially as the highest noise levels are higher than can be expected for real measurements.

Unlike one would expect there are no ghost peaks that appear in the 2D distributions far away from the expected solution space (Supplementary Figure 17). As can be seen in Supplementary Figure 19, the 1D distributions for the input to the simulation and for the output of the analysis still match very well. This is certainly due to the high spectral resolution and the confined solution space that is provided by the MWL-AUC and the OBC-method.

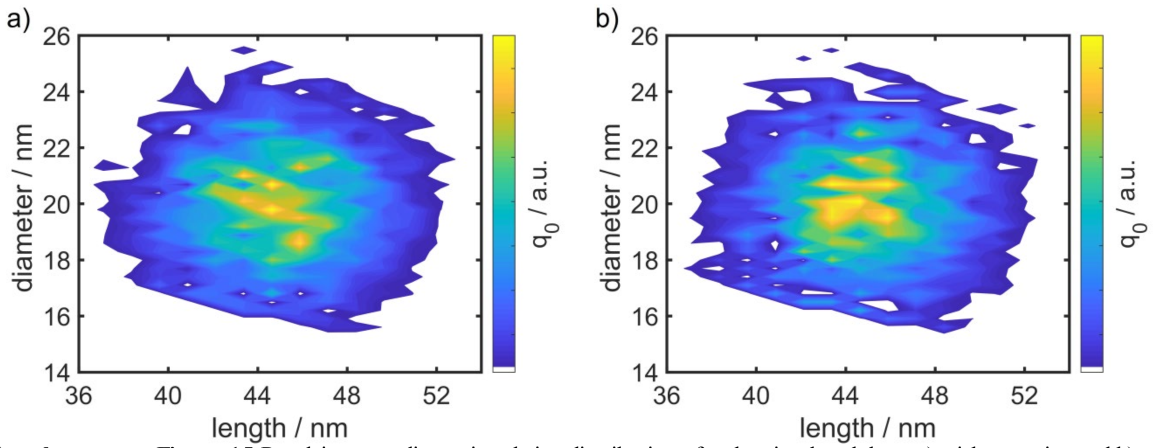

Supplementary Figure 15 Resulting two-dimensional size distributions for the simulated data, a) without noise and b) with a signal to noise ratio of 40 dB (right).

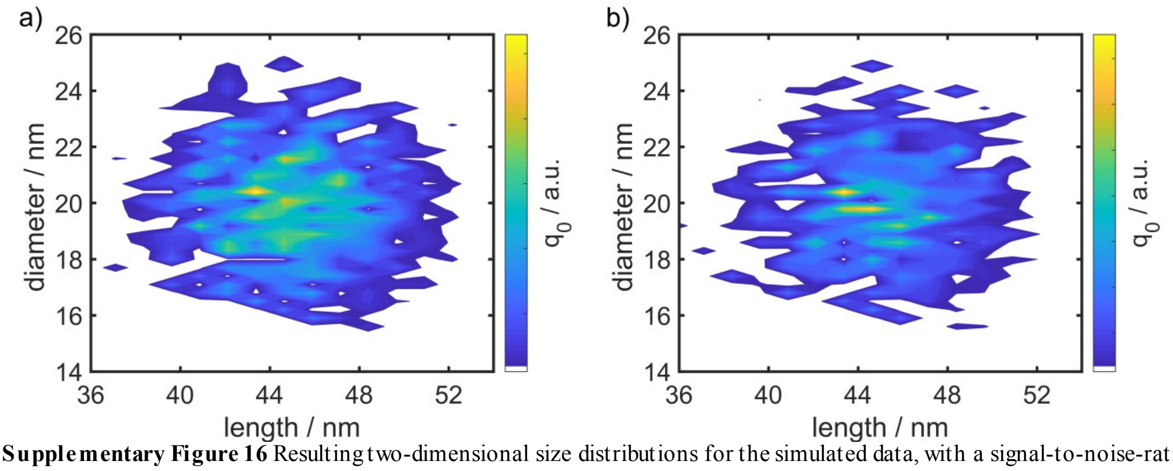

Supplementary Figure 16 Resulting two-dimensional size distributions for the simulated data, with a signal-to-noise-ratio of a) 20 and b) 10 dB.

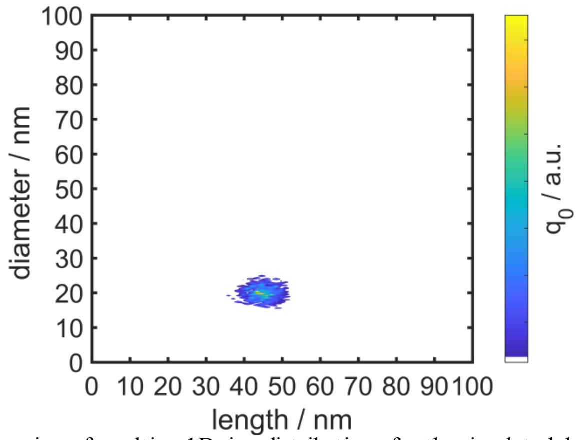

Supplementary Figure 17 Overview of resulting 1D size distributions for the simulated data with a signal-to-noise-ratio of 10 dB.

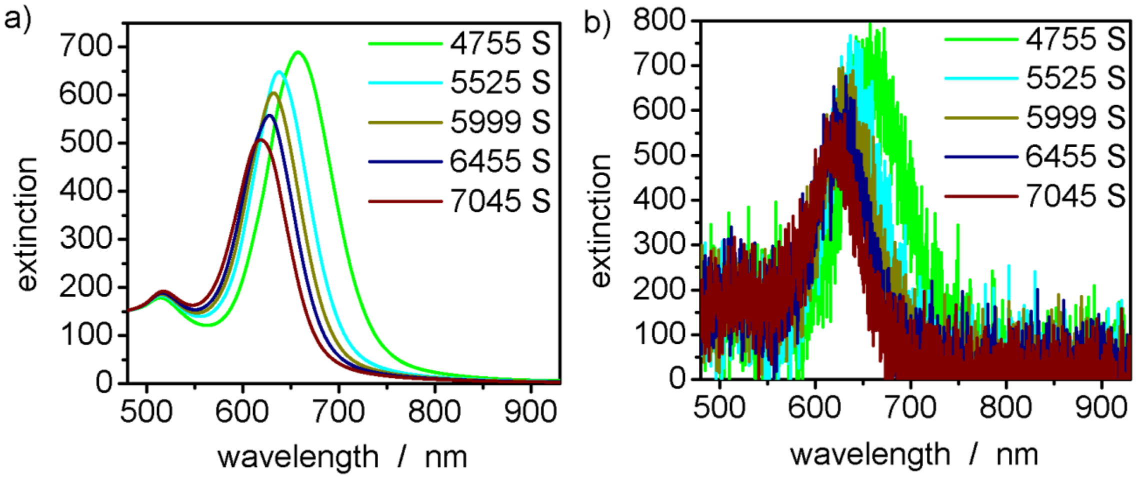

Supplementary Figure 18 Effect of gaussian noise on extracted spectra. Typical spectra extracted for the analysis for the simulated data a) without and b) with added noise (right, signal-to-noise-ratio 10dB).

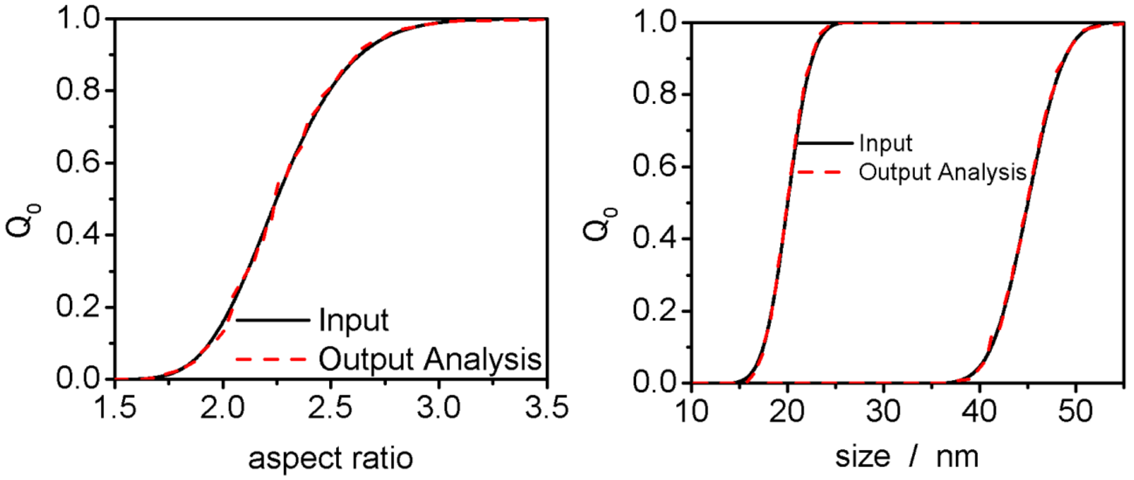

Supplementary Figure 19 Input and output one-dimensional distributions for the simulated data with added noise (signal-to-noise-ratio 10dB).

Supplementary Note 8: Non-ideality of sedimentation

For highly concentrated systems, non-ideality of sedimentation may be present. However, this is not applicable to the measured samples as the volume fraction is small, while the theoretical particle-particle distance is large. The volume fractions determined via the Mie-Gans model for the different samples are given in Supplementary Table 1. Results from ICP-MS measurements delivered by the supplier of sample Citrate 1 and 2 give a gold mass concentration of 0.03 and 0.018 mg/ml. This results in gold nanorod volume fractions of the diluted samples of $1.3 \cdot 10^{-6}$ and $7.8 \cdot 10^{-7}$. These volume fractions can be considered to be sufficiently small.

For the discussion of interactions it is useful to consider the theoretical distances between the particles under considerations. Using the particle concentrations given by the Gans theory, the volume per particle can be deduced. The particle-particle distance can then be calculated assuming the unit cell of the body-centered cubic lattice for the particles in solution. The

particle-particle distance given in Supplementary Table 1 can be considered to be much higher than the typical Debye length that one would even expect for low ionic strengths.

Supplementary Table 1 Concentration and particle-particle distances calculated from results of the OBC-method and the Gans model.

Sample	CTAB 1	CTAB 2	CTAB 3	Citrate 1	Citrate 2
Volume fraction / 10^{-7}	7.2	6.6	6.1	8.3	5.3
Concentration / $10^{16}\mathrm{m}^{-3}$	4.2	3.5	12	8.9	11
Solvent volume per particle / $10^{-18}\ \mathrm{m}^3$	24	28	8.1	11	9.5
Particle-particle distance / µm	3.1	3.3	2.2	2.4	2.3

Supplementary Note 9: Interpolation of simulated extinction spectra.

As outlined in the main part of this article, a problem-specific interpolation model for the approximation of extinction spectra of gold nanorods is established on the basis of highly accurate finite element simulation. In the following we provide more insight about how this model is generated and discuss its quality in more detail.

In order to be able to approximate the extinction spectra $\sigma_{\mathrm{ext}}(d,l;\lambda)$ with high accuracy for a broad range of geometric parameters d,l and wavelengths λ while keeping the number of finite-element-simulations low, the interpolation model is generated by an adaptive multistage process. We first define a non-uniform interpolation grid with respect to the geometric variables d,l with a maximal grid size of 5.4 nm. Then, for each grid point (d,l), we evaluate $\sigma_{\mathrm{ext}}(d,l;\lambda)$ for different choices of $\lambda \in [350\ \mathrm{nm}, 1250\ \mathrm{nm}]$. Instead of using a fixed grid for the wavelength discretization, an adaptive method is applied, which guarantees that for each interior gridpoint λ_i the condition

$$\frac{\left|\sigma_{\mathrm{ext}}(\lambda_i)-\frac{\lambda_i-\lambda_{i-1}}{\lambda_{i+1}-\lambda_{i-1}}\sigma_{\mathrm{ext}}(\lambda_{i-1})-\frac{\lambda_{i+1}-\lambda_i}{\lambda_{i+1}-\lambda_{i-1}}\sigma_{\mathrm{ext}}(\lambda_{i+1})\right|}{\sigma_{\mathrm{ext}}(\lambda_i)}<1\% \qquad (19)$$

is satisfied (note that to unburden notation we have omitted the dependence on d and l here) or the local grid size given by the formula $\max\{|\lambda_{i+1}-\lambda_i|,|\lambda_{i+1}-\lambda_i|\}$ is below 1 nm. Both conditions constitute a rigorous error bound provided that the function $\sigma_{\mathrm{ext}}(d,l;\lambda)$ is at least twice continuously differentiable with respect to λ. Now, by piecewise cubic interpolation, we obtain a high fidelity model $\overline{\sigma}_{\mathrm{ext}}(d,l;\lambda)$ which can be evaluated for arbitrary wavelengths as

well as each grid point (d, l). Next, we want to establish an interpolation model with respect to the (d, l)-grid. For this, we first decompose $\sigma_{\text{ext}}(d, l; \lambda)$ in the following way:

$$\sigma_{\text{ext}}(d, l; \lambda) = \sigma_{\text{ext}}^{\text{fit}}(d, l; \lambda) + \sigma_{\text{ext}}^{\text{res}}(d, l; \lambda) \tag{20}$$

$$\sigma_{\text{ext}}^{\text{fit}}(d, l; \lambda) := \frac{a(d, l)}{b(d, l)\big(\lambda. -c(d, l)\big)^2 + 1} \tag{21}$$

$$\sigma_{\text{ext}}^{\text{res}}(d, l; \lambda) := \sigma_{\text{ext}}(d, l; \lambda) - \sigma_{\text{ext}}^{\text{fit}}(d, l; \lambda). \tag{22}$$

Here, the first term $\sigma_{\text{ext}}^{\text{fit}}(d, l; \lambda)$ is chosen to capture the characteristics of the principal peak in the spectrum and is given in terms of three shape dependent coefficients $a(d, l), b(d, l)$ and $c(d, l)$, which are closely related to the height, width and position of the peak, respectively. Next, for each pair (d, l) these coefficients are determined by minimizing the squared distance between the interpolation model $\overline{\sigma}_{\text{ext}}(d, l; \lambda)$ and $\sigma_{\text{ext}}^{\text{fit}}(d, l; \lambda)$ on the λ-interval $[600\text{ nm}, 1250\text{ nm}]$. As each of these minimization problems is defined only in three optimization variables, global minima can be calculated in all cases with a reasonable computational effort. Exemplarily, the interpolation model as well as the decomposition of the spectrum $\sigma_{\text{ext}}(d, l; \lambda)$ using the optimized coefficients $a(d, l)$, $b(d, l)$ and $c(d, l)$ is visualized in Supplementary Figure 20 for four selections of d and l. Now, a, b and c are interpolated by a standard piecewise cubic interpolation scheme; while a and c are directly interpolated in the parameters d and l, for b a reparameterization in terms of aspect ratio and diameter provides better results. The resulting functions are depicted in Supplementary Figure 21. It is observed that a, b and c are rather smooth with respect to the geometric parameters d and l. The final step of the approximation scheme is to interpolate the peak function $\sigma_{\text{ext}}^{\text{fit}}(d, l; \lambda)$ itself as well as the remainder term $\sigma_{\text{ext}}^{\text{res}}(d, l; \lambda)$ with respect to the (d, l)-grid. It is noted that, in contrast to $\sigma_{\text{ext}}^{\text{fit}}(d, l; \lambda)$ a standard interpolation for $\sigma_{\text{ext}}^{\text{res}}(d, l; \lambda)$ appears to be sufficient, as for the part of the spectrum captured by $\sigma_{\text{ext}}^{\text{res}}(d, l; \lambda)$ variations of d and l show mainly a scaling effect rather than a shift in the peak position, compare Supplementary Figure 20.

We now want to discuss the quality of our nonlinear interpolation model $\sigma_{\text{ext}}^{\text{interp}}$. We do that in two different ways. First, we evaluate the final interpolation function at points (d, l) which were not contained in the (d, l)-grid used for the interpolation process and compare the obtained extinction spectrum to the spectrum obtained from a highly accurate FE simulation. The general observation is that the interpolation model matches the FE results very well. Exemplarily this is demonstrated in Supplementary Figure 22 and Supplementary Figure 22 for two arbitrarily selected choices of d and l. To justify our approximation process which

appears to be slightly more complicated compared to standard interpolation schemes, we further compare $\sigma_{\mathrm{ext}}^{\mathrm{interp}}$ to a piecewise linear interpolation model $\sigma_{\mathrm{ext}}^{\mathrm{interp,lin}}$ which directly interpolates $\sigma_{\mathrm{ext}}(d, l; \lambda)$ on the (d, l) -grid, see Supplementary Figure 22 as well as Supplementary Figure 23. It is observed that the standard interpolation causes artificial „double-peaks" in the spectrum, which would clearly lead to artifacts in the derived particle size distributions. Remarkably, the occurrence of the double-peaks turns out to be independent of the chosen order of the interpolation scheme. Of course, one could enforce these artifacts to disappear by choosing a very fine (d, l)-grid, however, our numerical experiments reveal that a reasonable approximation is achieved only at a grid size below 0.1 nm leading to an intractable number of finite element simulations.

In conclusion, we were able to establish on the basis of an affordable number of finite element solutions a very accurate interpolation model for the extinction spectra $\sigma_{\mathrm{ext}}(d, l; \lambda)$ of gold nanorods. This model can be evaluated for arbitrary wavelengths as well as choices of geometric parameters in the given ranges without the need of further finite element simulations. Furthermore, the described approach can be applied to derive cheap and accurate models of any kind of particle shape which can be described by a reasonably small number of parameters.

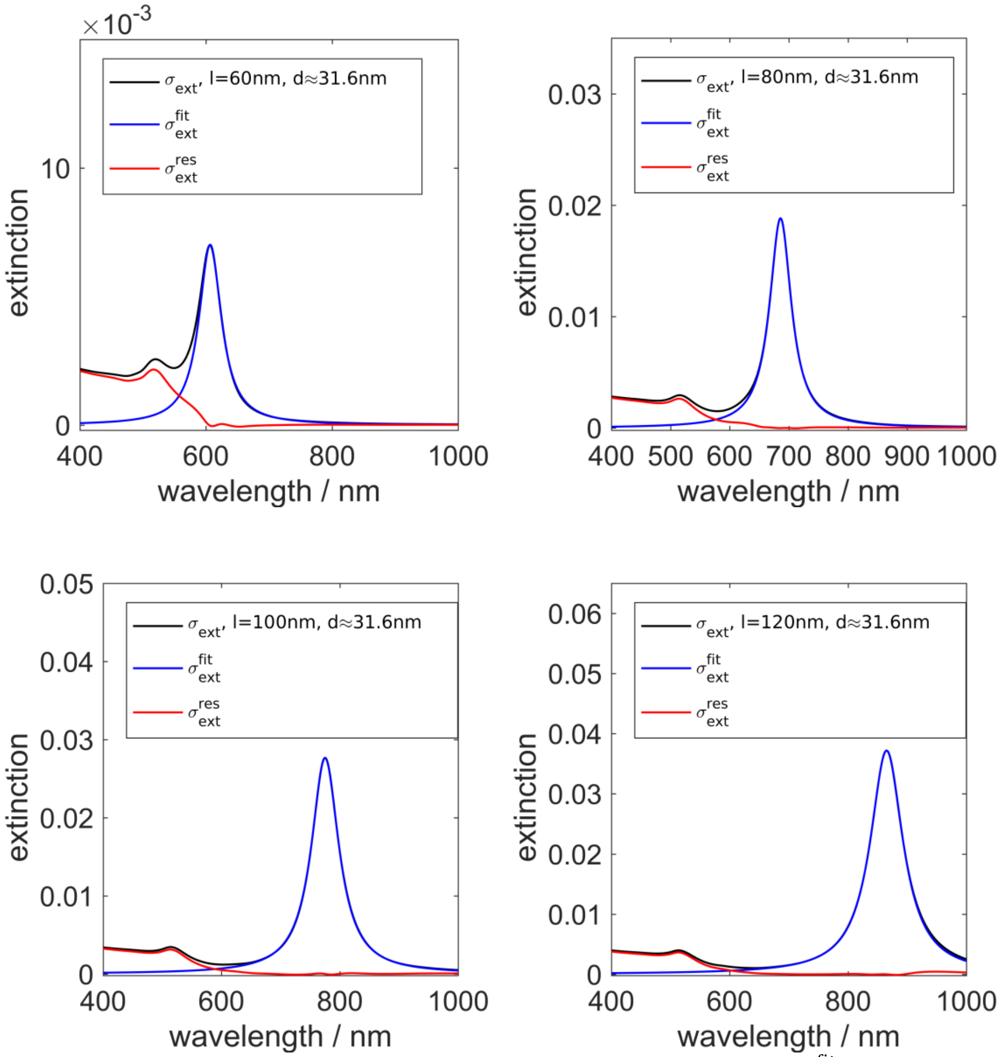

Supplementary Figure 20 Exemplarily, the decomposition of the spectrum $\sigma_{\text{ext}}(d,l;\lambda)$ (black) into $\sigma_{\text{ext}}^{\text{fit}}(d,l;\lambda)$ (blue) and $\sigma_{\text{ext}}^{\text{res}}(d,l;\lambda)$ (red) is illustrated for four selected pairs of d and l.

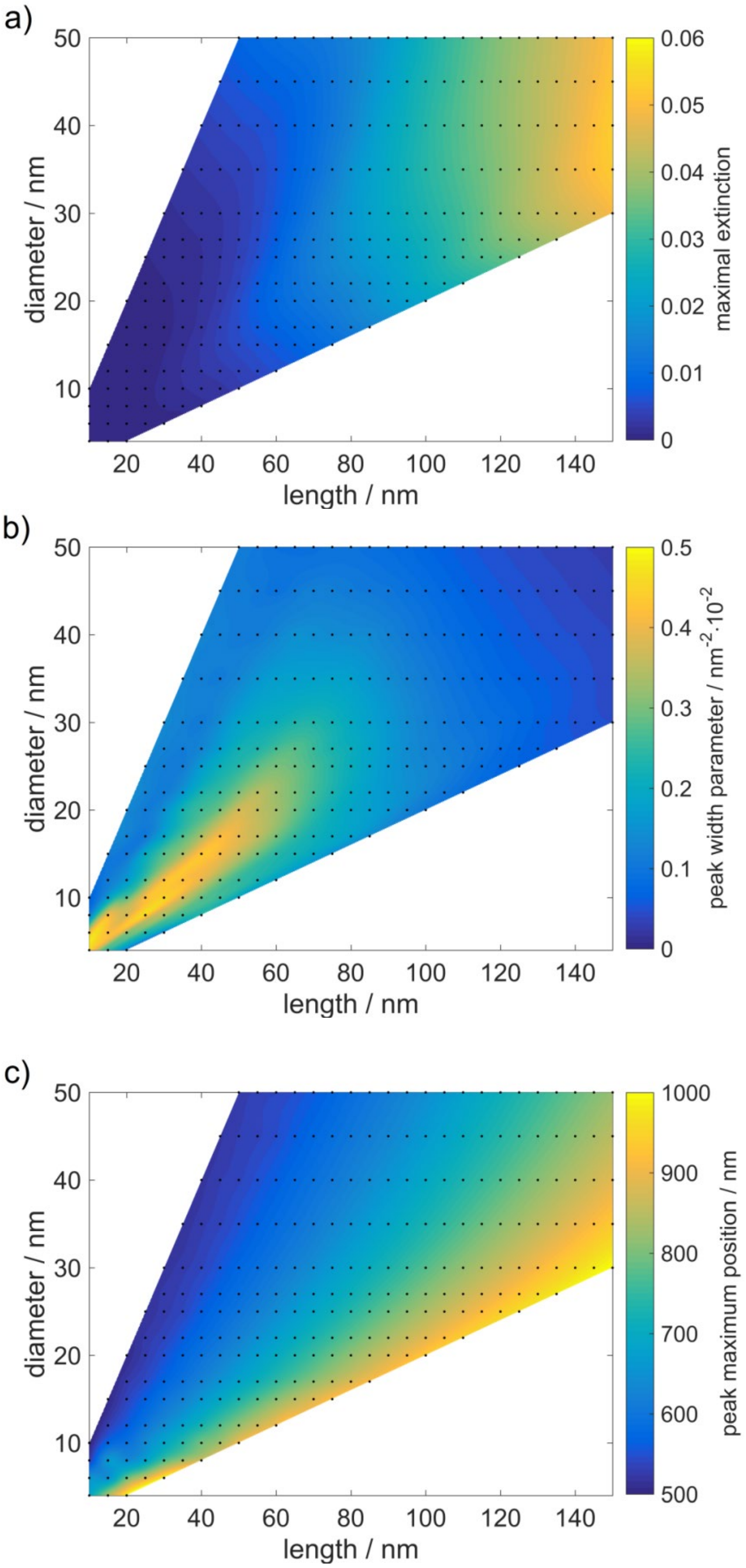

Supplementary Figure 21 The coefficients a, b and c are displayed for all admissible values of d and l. a) The coefficient a represents the height of the principle peak in the spectrum; as expected, an increase of the peak is observed for increasing values of d and l. b) The coefficient b represents the peak width and c) the coefficient c represents the peak position. All interpolated functions show a smooth dependence on d and l.

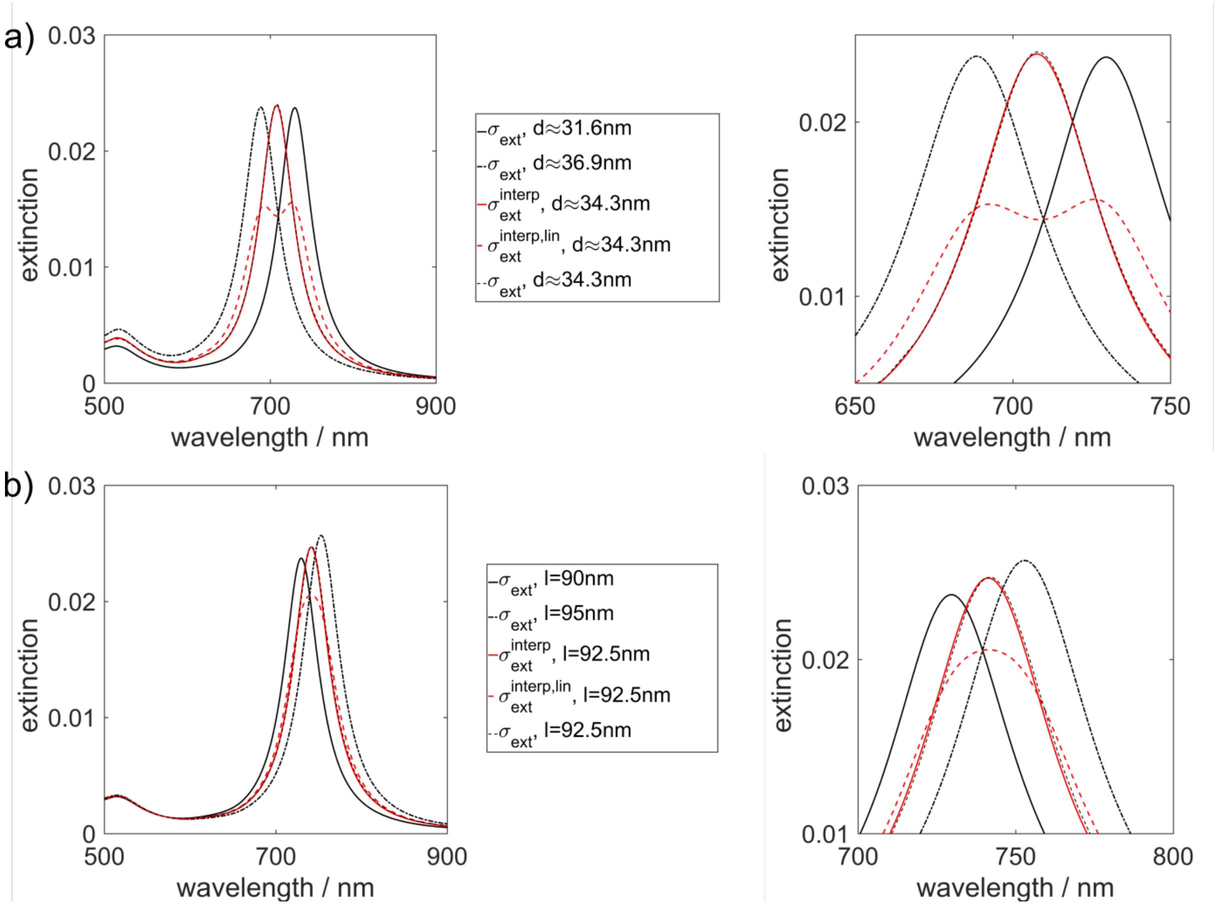

Supplementary Figure 22 Our interpolation model $\boldsymbol{\sigma}_{\mathbf{ext}}^{\mathbf{interp}}$ is compared to a "standard" linear interpolation model $\boldsymbol{\sigma}_{\mathbf{ext}}^{\mathbf{interp,lin}}$. a) The standard interpolation model $\boldsymbol{\sigma}_{\mathbf{ext}}^{\mathbf{interp,lin}}$ gives rise to artificial double-peaks, which do not occur in the graph of $\boldsymbol{\sigma}_{\mathbf{ext}}^{\mathbf{interp}}$. In a) we have used a fixed length $\boldsymbol{l} = \mathbf{90\ nm}$ and a variable diameter $\boldsymbol{d} \in [\mathbf{31.6\ nm}, \mathbf{36.9\ nm}]$ in b) the diameter is fixed to $\boldsymbol{d} \approx \mathbf{31.6\ nm}$ and the length is chosen as $\boldsymbol{l} \in [\mathbf{90\ nm}, \mathbf{95\ nm}]$. Moreover it is demonstrated that a comparison of our model evaluated for a point not contained in the $(\boldsymbol{d}, \boldsymbol{l})$ -grid (red solid line) to a the spectrum obtained from a highly accurate finite element simulation (black dashed line) shows an excellent match.

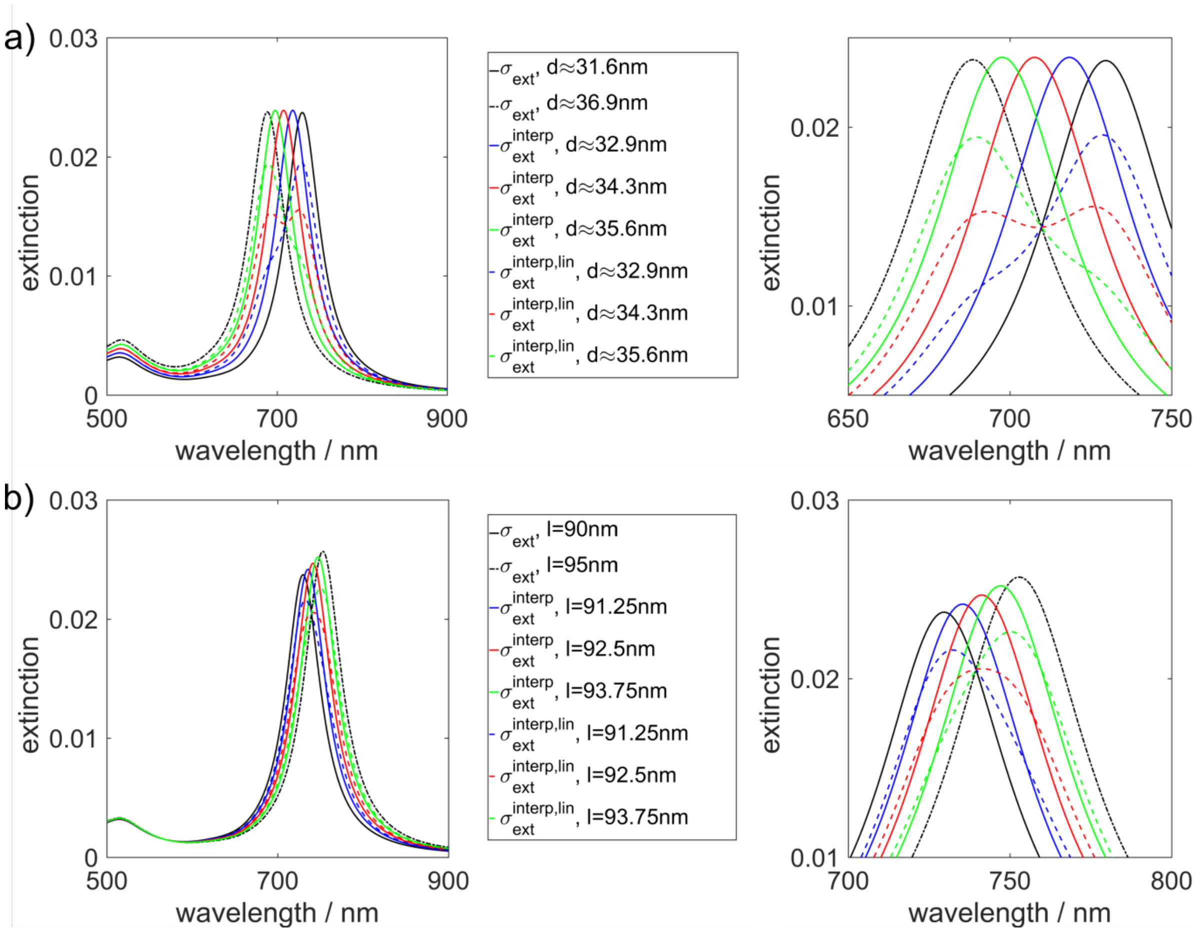

Supplementary Figure 23 Example of spectral interpolation. Similar to Supplementary Figure 22 the models $\boldsymbol{\sigma}_{\mathbf{ext}}^{\mathbf{interp}}$ and $\boldsymbol{\sigma}_{\mathbf{ext}}^{\mathbf{interp,lin}}$ are compared for different choices of $\boldsymbol{l}$ and $\boldsymbol{d}$. In a) we have used a fixed length $\boldsymbol{l} = \mathbf{90\ nm}$ a variable diameter $\boldsymbol{d} \in [\mathbf{31.6\ nm}, \mathbf{36.9\ nm}]$, in b) the diameter is fixed to $\boldsymbol{d} \approx \mathbf{31.6\ nm}$ and the length is chosen as $\boldsymbol{l} \in [\mathbf{90\ nm}, \mathbf{95\ nm}]$.

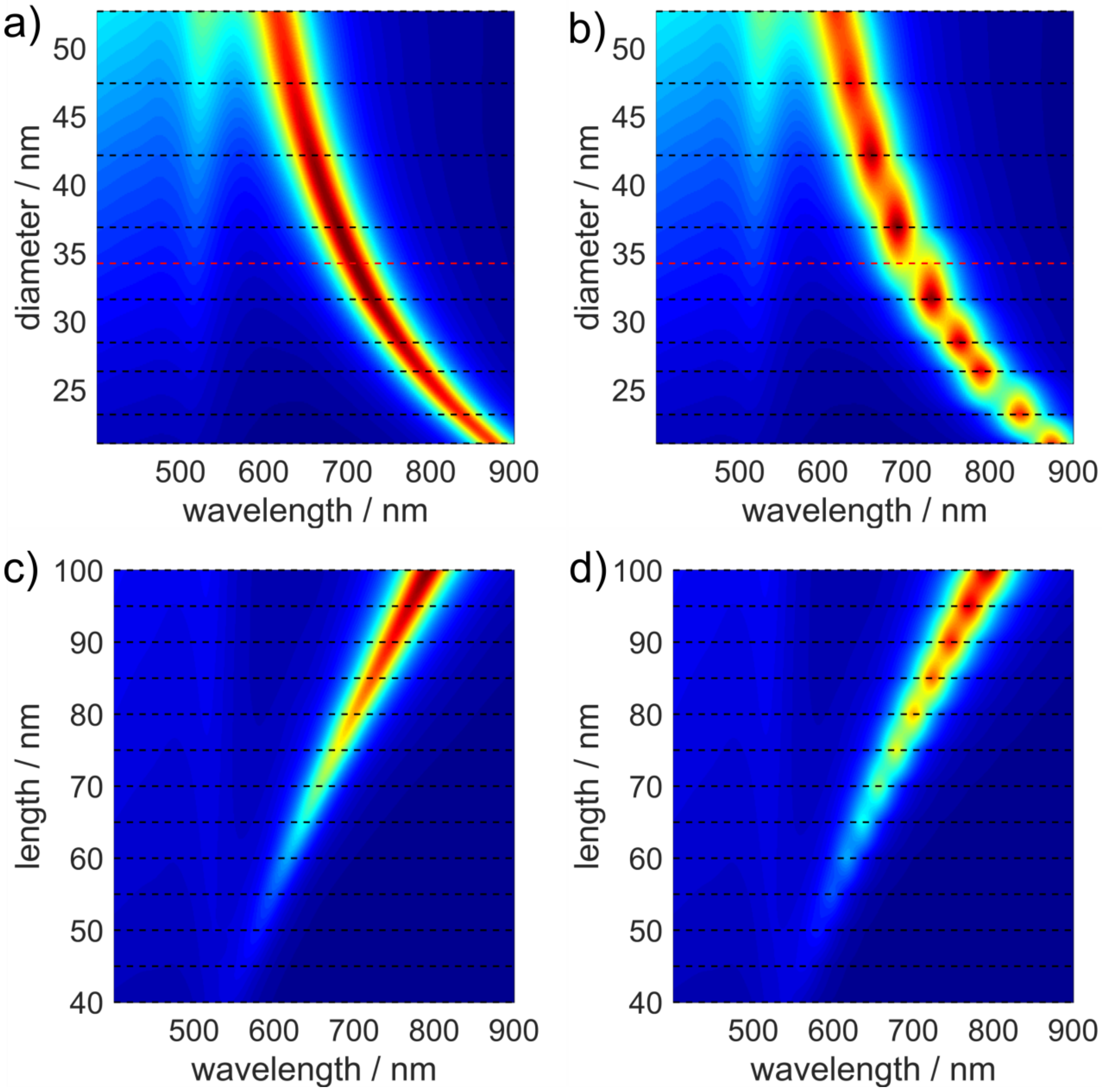

Supplementary Figure 24 Comparison of the interpolation approaches. The spectra for a wide range of diameters for fixed length ($l = 90$ nm, a) and b)) and vice versa ($d \approx 31.6$ nm, c) and d)) are displayed for the classical linear interpolation scheme $\sigma_{\text{ext}}^{\text{interp,lin}}$ (right column) and the one developed within this article $\sigma_{\text{ext}}^{\text{interp}}$ (a) and c)). The dashed red lines in the a) and b) indicate the choice of d and l for which the “double-peak” occurs in the linear interpolation setting, see also the red dashed curve in Supplementary Figure 22 (a)). The black-dashed lines indicate parameters for which the extinction cross section has been computed numerically while in-between the values were interpolated based on the numerical simulations.

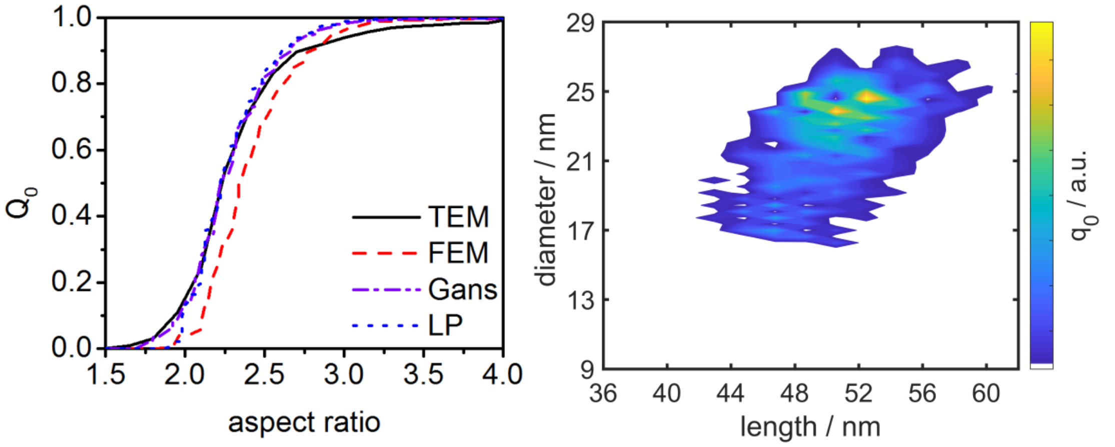

Supplementary Figure 25 One-dimensional aspect ratio distribution for the different optical models and 2D distribution for the LP model for the first CTAB sample.

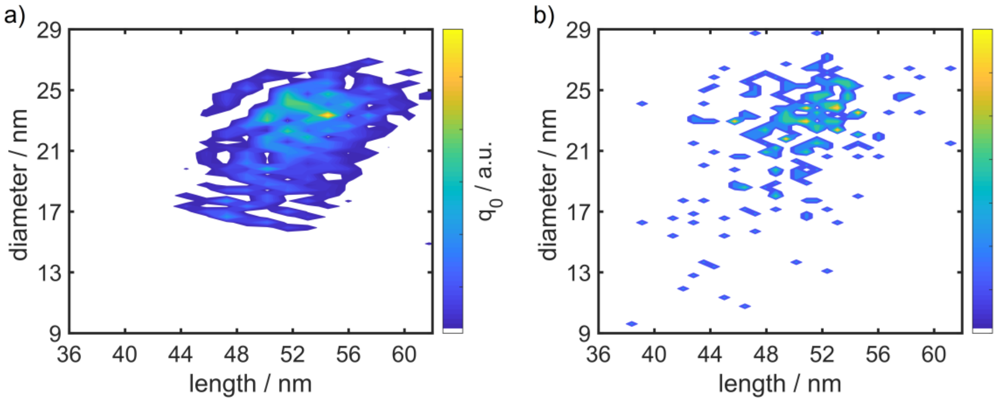

Supplementary Figure 26 2D size distributions of the first CTAB sample. a) shows the OBC FEM and b) the TEM distribution of length and diameter.

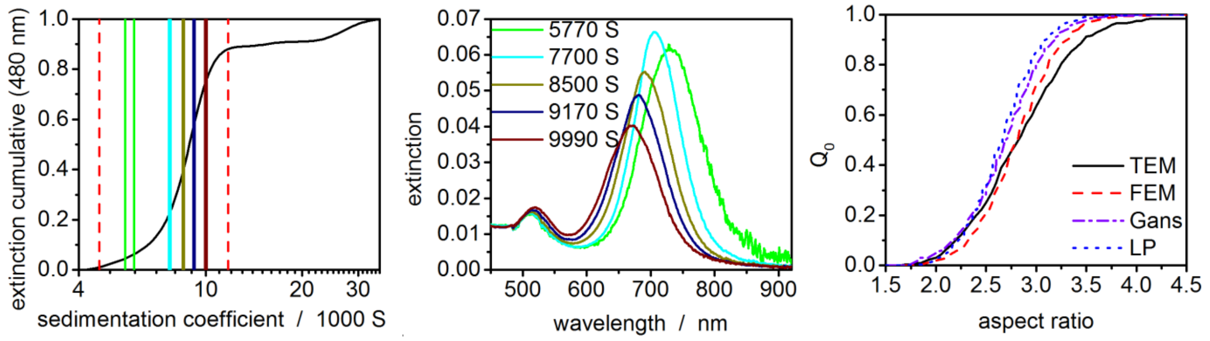

Supplementary Figure 27 Sedimentation intervals, extracted spectra and resulting aspect ratio distribution with mean sedimentation coefficients for the second CTAB sample. For reasons of clarity only selected spectra and intervals are shown. The color of the intervals corresponds to the upper and lower boundary as well as to the extracted spectra. The interval length varies according to the cumulative distribution from 70 to 570 S.

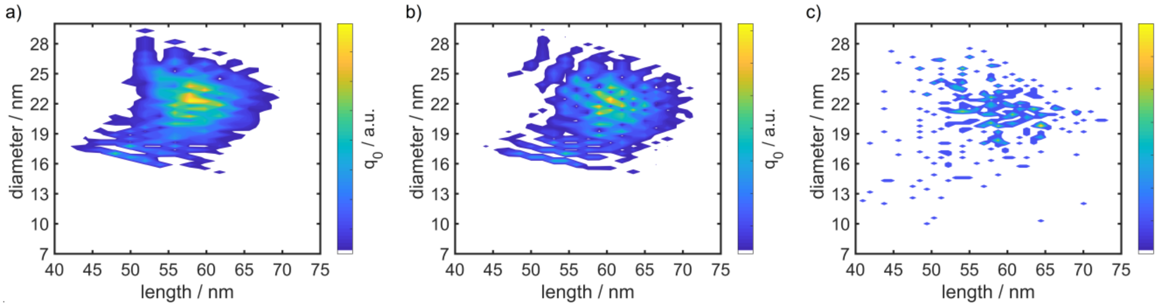

Supplementary Figure 28 2D size distributions of the second CTAB sample. a) shows the OBC-AUC LP, b) the OBC-AUC FEM and c) TEM distribution of length and diameter.

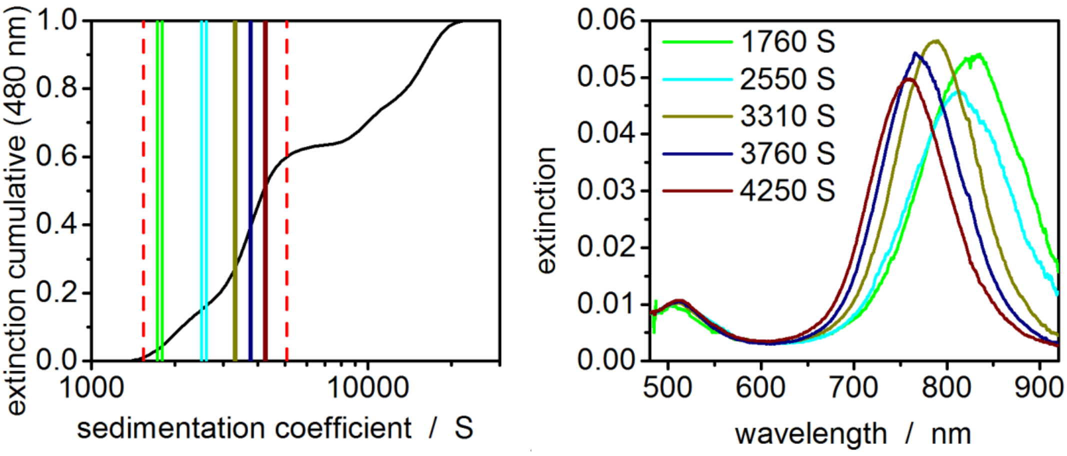

Supplementary Figure 29 Sedimentation intervals and extracted spectra for the third CTAB sample. For reasons of clarity only selected spectra and intervals are shown. The color of the intervals corresponds to the upper and lower boundary as well as to the extracted spectra. The interval length varies according to the cumulative distribution from 40 to 340 S.

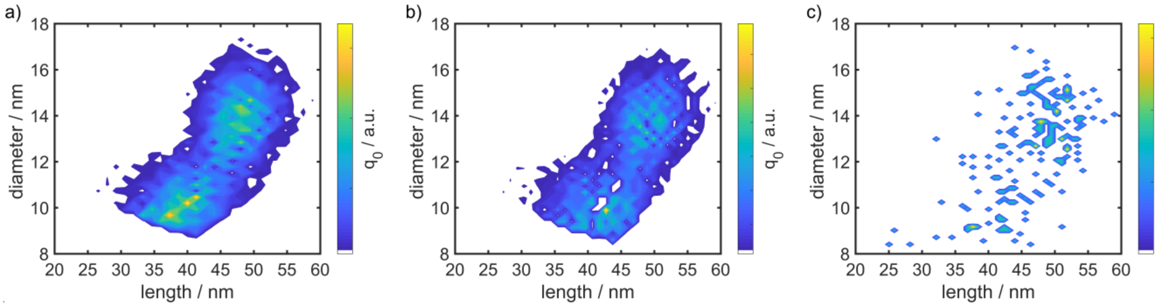

Supplementary Figure 30 2D size distributions of the third CTAB sample. a) shows the OBC-AUC LP, b) the OBC-AUC FEM and c) TEM distribution of length and diameter.

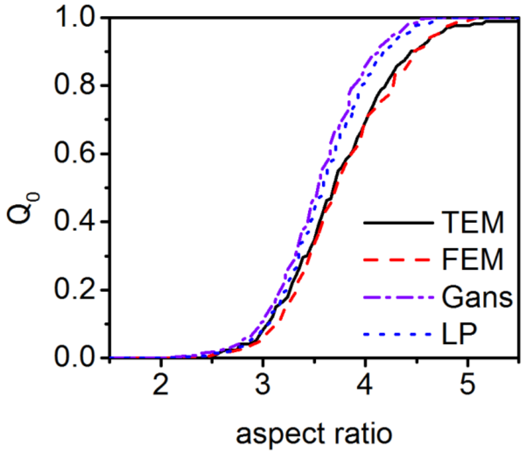

Supplementary Figure 31 Aspect ratio distributions of CTAB sample 3. The resulting one-dimensional aspect ratio distributions measured with TEM and AUC using the different optical models are compared.

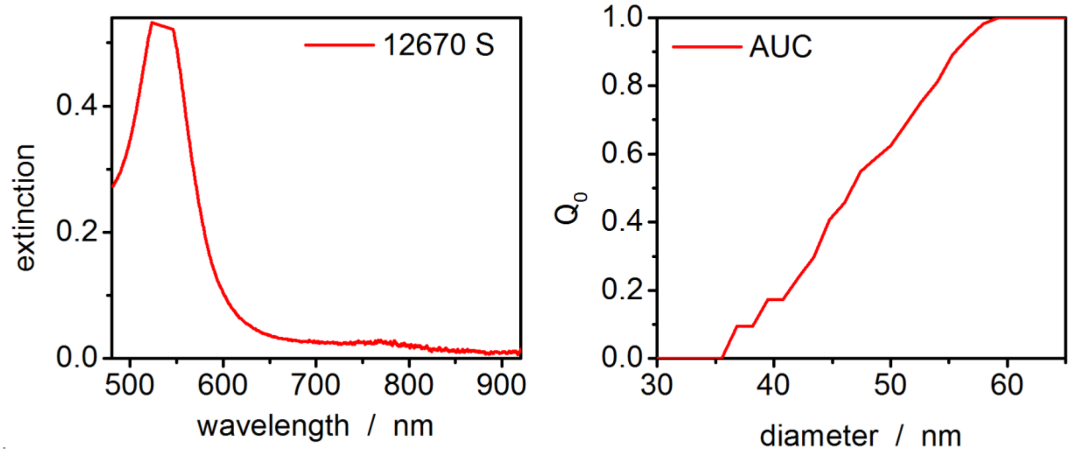

Supplementary Figure 32 Spectra and size distribution of gold nanospheres. Extracted spectra were deduced from CTAB sample 3 in the range of 0.61 - 0.99 of the cumulative sedimentation coefficient distribution with mean sedimentation coefficient, indicating the presence of large amounts of gold nanospheres and respective particle size distribution for 25 sedimentation coefficient intervals.

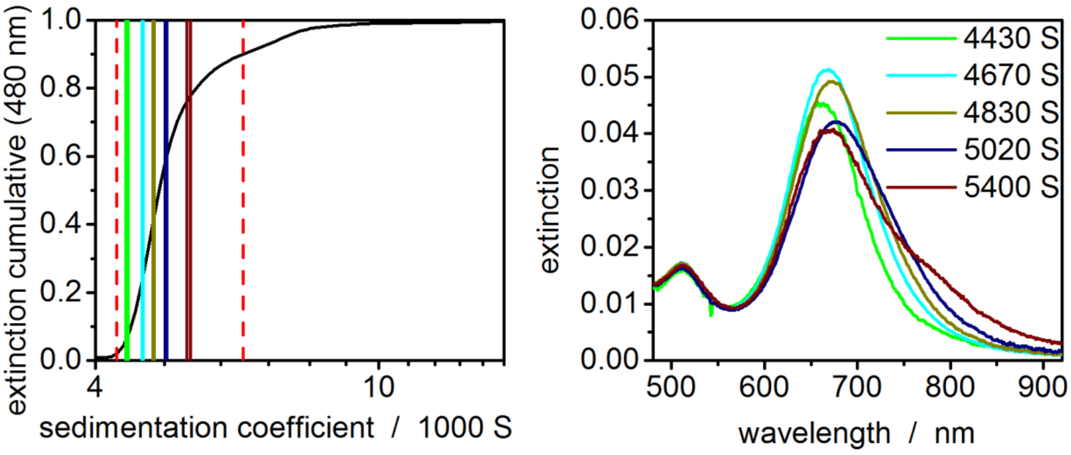

Supplementary Figure 33 Sedimentation intervals and extracted spectra with mean sedimentation coefficients for citrate sample 1. For reasons of clarity only selected spectra and intervals are shown. The color of the intervals corresponds to the upper and lower boundary as well as to the extracted spectra. The interval length varies according to the cumulative distribution from 20 to 280 S.

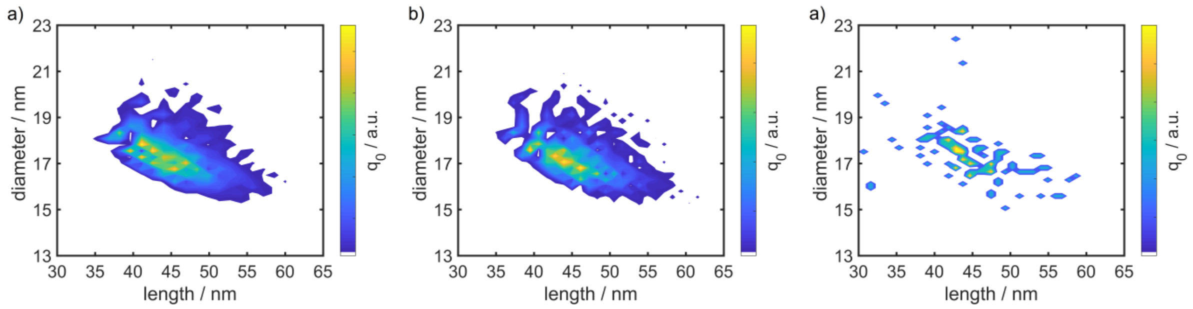

Supplementary Figure 34 2D size distributions of the first citrate sample. a) shows the OBC-AUC LP, b) the OBC-AUC FEM and c) TEM distribution of length and diameter.

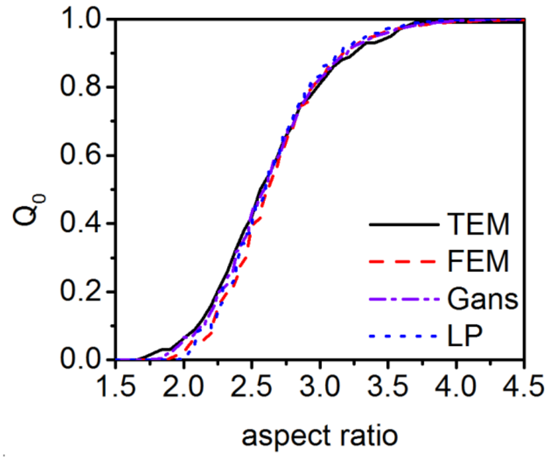

Supplementary Figure 35 Results for aspect ratio after determining the relevant parameters for the different optical models and TEM distributions for the first citrate sample.

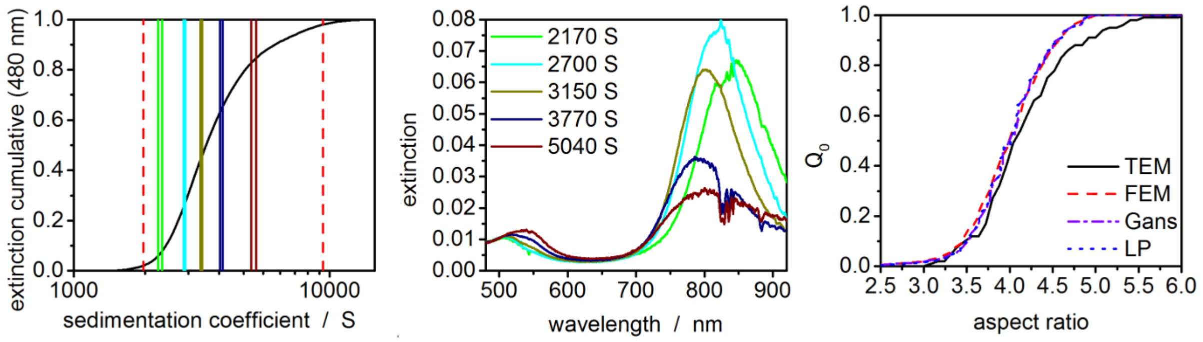

Supplementary Figure 36 Sedimentation intervals, extracted spectra with mean sedimentation coefficients and aspect ratio distribution for the different optical models for the second citrate sample. For reasons of clarity only selected spectra are shown. The color of the intervals corresponds to the upper and lower boundary as well as to the extracted spectra. The interval length varies according to the cumulative distribution from 20 to 180 S.

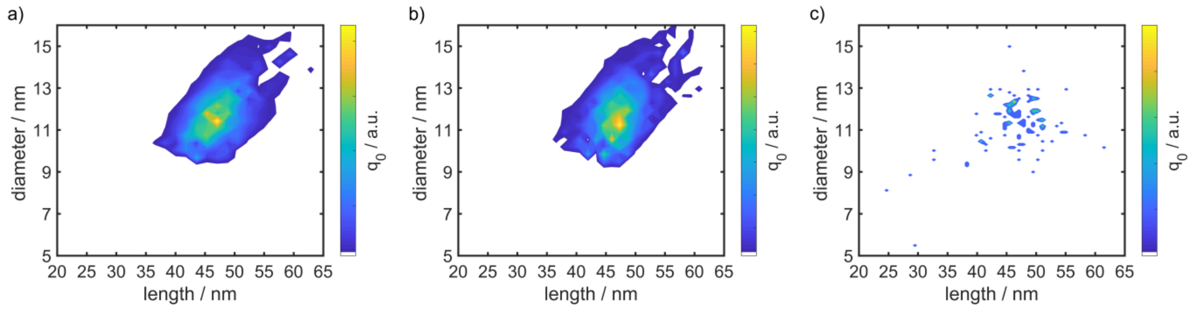

Supplementary Figure 37 Two-dimensional size distributions of the second citrate sample. OBC-AUC results using a) LP, b) FEM models and c) TEM distribution of length and diameter.

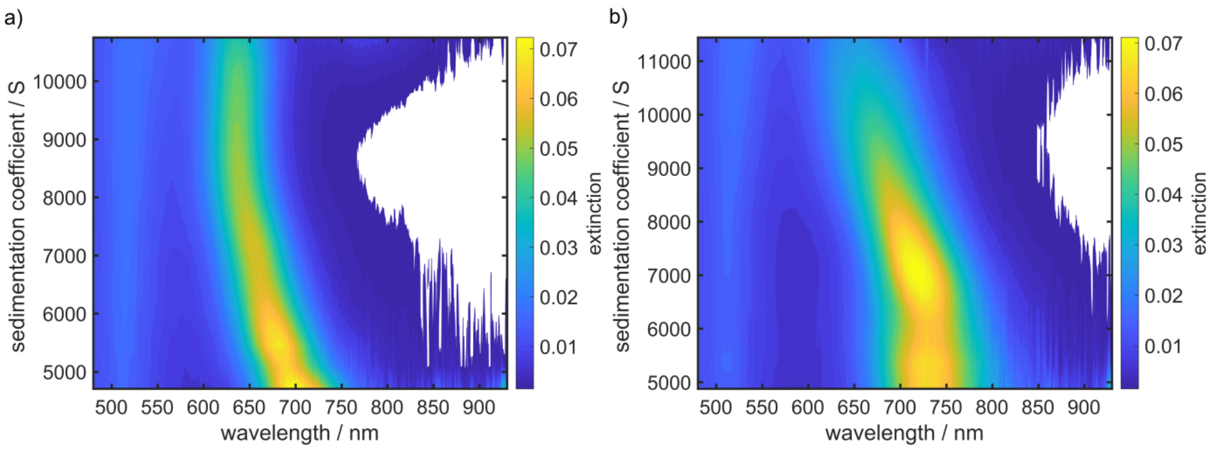

Supplementary Figure 38 MWL-AUC raw data. Spectral and sedimentation data for a) CTAB sample 1 and b) CTAB sample 2 used for analysis. Please note that the extinction is based on the natural logarithm.

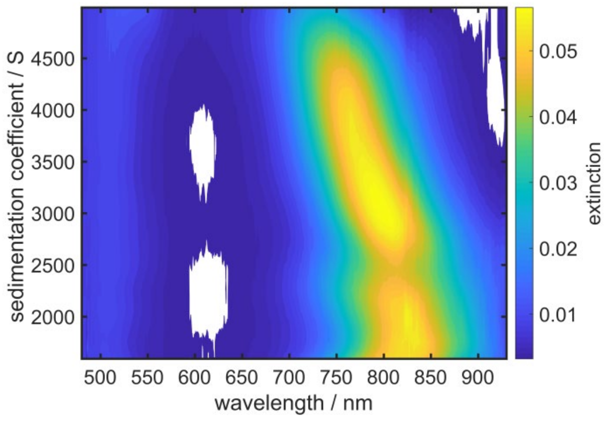

Supplementary Figure 39 MWL-AUC raw data. Spectral and sedimentation data for CTAB sample 3 used for analysis. Please note that the extinction is based on the natural logarithm.

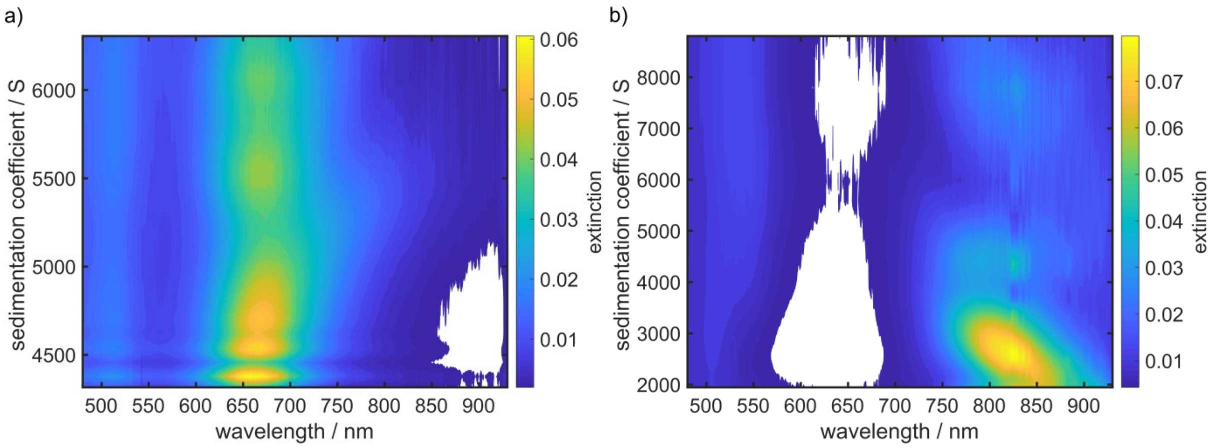

Supplementary Figure 40 MWL-AUC raw data. Spectral and sedimentation data for a) citrate sample 1 and b) citrate sample 2 used for analysis. Please note that the extinction is based on the natural logarithm.

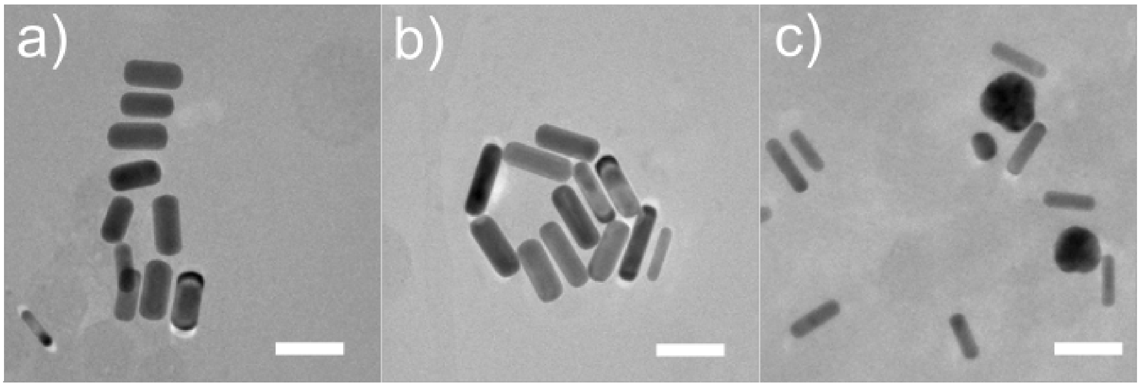

Supplementary Figure 41 Typical TEM image of CTAB samples a) one, b) two and c) three. The scale bar is 60 nm for all the samples

Supplementary references

1. Olson, J. *et al.* Optical characterization of single plasmonic nanoparticles. *Chemical Society reviews* 44, 40–57 (2015).
2. Gans, R. Über die Form ultramikroskopischer Goldteilchen. *Annalen der Physik* 342, 881–900 (1912).
3. Link, S., Mohamed, M.B. & El-Sayed, M.A. Simulation of the Optical Absorption Spectra of Gold Nanorods as a Function of Their Aspect Ratio and the Effect of the Medium Dielectric Constant. *J. Phys. Chem. B* 103, 3073–3077 (1999).
